# Strength-ductility synergy in medium-entropy alloys via harnessing trace air in additive manufacturing

**DOI:** 10.1038/s41467-026-72511-8

**Published:** 2026-04-29

**Authors:** Yansheng Li, Jiawei Yin, Meiyuan Jiao, Tengfei Zheng, Yuan Wu, Shimiao Li, Guohui Zhang, Jiabin Yu, Yunzhuo Lu, Chun Shang, Haiou Yang, Yang He, Huihui Zhu, Sheng Zhang, Xiaobin Zhang, Xiongjun Liu, Suihe Jiang, Hui Wang, Zhaoping Lu

**Affiliations:** 1 State Key Laboratory for Advanced Metals and Materials, University of Science and Technology Beijing, Beijing, China; 2 Research Institute of Advanced Materials (Shenzhen) Co., LTD, China Iron & Steel Research Institute Group, Shenzhen, China; 3 School of Mathematics and Physics, University of Science and Technology Beijing, Beijing, China; 4 School of Materials Science and Engineering, Dalian Jiaotong University, Dalian, China; 5 State Key Laboratory of Solidification Processing, Northwestern Polytechnical University, Xi’an, China; 6 Beijing Advanced Innovation Center for Materials Genome Engineering, School of Materials Science and Engineering, University of Science and Technology Beijing, Beijing, China; 7 School of Mechanical Engineering, University of Science and Technology Beijing, Beijing, China

**Keywords:** Metals and alloys, Mechanical properties

## Abstract

Conventional additive manufacturing (AM) of metallic materials demands costly high-vacuum or ultra-pure inert atmospheres to suppress impurity-induced embrittlement. Here, we overturn this paradigm by demonstrating that ambient trace O and N in an inert atmosphere can be turned into potent in-situ alloying species so that the strength and ductility of the material can be simultaneously enhanced. In a Ti_56_Zr_30_Nb_14_ medium-entropy alloy (MEA) additively manufactured with optimized air doping, the yield strength rises by 67% to ≈1 GPa and the tensile ductility increases by 64% to ≈18%, achieving a simultaneous gain that defies the classical strength-ductility trade-off. Atom-probe tomography, enhanced by a machine-learning workflow, identifies two distinct families of nanoscale ordered interstitial complexes (OICs): O-rich OIC1 (O-Zr-Ti) and N-rich OIC2 (N-Zr-Ti). These complexes act as potent dislocation-pinning sites while promoting extensive cross-slip of dislocations and activating Frank-Read sources during plastic deformation. The resultant wavy slip and sustained work-hardening capacity give rise to exceptional strength-ductility synergy. Eliminating the need for high-purity inert gas, this air-alloying route delivers a low-cost, scalable pathway to strong-yet-ductile AM metallic materials.

## Abstract (web summary)

Typically, metal additive manufacturing requires costly high vacuum/ultra-pure inert atmospheres to avoid material embrittlement. This alloy design simultaneously boosts a medium-entropy alloy’s strength and ductility through trace oxygen and nitrogen in-situ alloying, eliminating high-purity gas needs.

## Introduction

Additive manufacturing (AM) has emerged as a transformative technology for metallic materials, enabling net-shape fabrication of geometries that are impossible or prohibitively expensive to cast, forge, or machine^1,2^. In the biomedical arena, this capability proves revolutionary. Patient-specific implants such as mandibular segments, scapular plates, and pelvic reinforcements are fabricated directly from CT or MRI data, delivering an exact anatomical fit while eliminating costly, time-intensive custom tooling^3–5^. Beyond healthcare, the same advantages accelerate aerospace engine mounts, thick-walled gearbox housings, and on-demand spare shafts for remote installations^6^. By eliminating dies, reducing material waste, and condensing the design-to-part cycle from months to days, AM not only lowers life-cycle costs but also unlocks functional performance that conventional metallurgy cannot deliver^7^.

Nevertheless, AM technology is highly sensitive to residual impurity O and N atoms introduced during powder handling and melt-pool exposure; even ppm levels can embrittle the alloy and degrade mechanical performance^8,9^. Therefore, impurity contents in AM products must be held below an ultra-low threshold under rigorously controlled atmospheres^10^; only then can printed parts meet service-level mechanical demands^11^. Traditional mitigation relies on high-purity inert atmospheres or ultrahigh vacuum, dramatically raising capital and operating costs and limiting scalability. Consequently, a fundamental rethink of how impurities are controlled—and potentially leveraged—during AM is urgently needed to fully realize the industrial potential of this technology.

Recently, it was reported that medium-entropy alloys (MEA) TiZrNb have great potential to be utilized as biomedical implant materials due to their high corrosion resistance, full biocompatibility (i.e., non-toxicity), and appropriate elastic modulus match with bones^12–15^. Thus, rapidly developing robust AM protocols for TiZrNb MEAs has become an urgent imperative for their timely clinical translation and widespread commercial deployment. Yet both Ti and Zr have strong affinity for oxygen/nitrogen and high interstitial solubility, making them highly susceptible to O and N contamination during printing^9,16^. Particularly, these same alloys can host large amounts of atoms^17^, so the central challenge is to mitigate or even harness, rather than merely suppress, such impurities during AM processing.

Fortunately, it was found that oxygen incorporation in TiZrHfNb HEAs can induce the formation of nanosized “ordered oxygen complexes” whose energy state lies between random interstitials and oxides. These localized short-range order (SRO) structures simultaneously increase the strength and ductility by promoting cross-slip and dislocation multiplication while avoiding the embrittlement typically associated with interstitial atoms^18^. Similar effects have been observed in TiZrNb-based MEAs and FeCoNiCrMn HEAs^19–23^, hinting at a route to turn contamination into an advantage. However, whether such special SROs form in AM-produced TiZrNb MEAs under trace-air-doping conditions and whether their structure and properties can be tuned by the air-doping level remain entirely unexplored.

In this study, we investigated AM processing of a Ti_56_Zr_30_Nb_14_ (at.%) MEA via the laser-directed energy deposition (L-DED) technique with deliberate air doping. Fully dense and crack-free parts were reproducibly obtained, permitting systematic evaluation of how incorporated O and N dictate microstructure and mechanical response. At an optimized air-doping level, an unusual simultaneous improvement in both strength and plasticity was achieved, with the yield strength exceeding 1 GPa and the plasticity surpassing 18%. Coupling advanced characterization with machine learning (ML) revealed two nanoscale oxygen/nitrogen-stabilized short-range order (SRO) motifs (hereafter referred to as OICs). These OICs reconfigure dislocation motion and work-hardening, converting atmospheric impurities into potent in situ alloying agents, opening a scalable path to strong yet ductile AM metals without the cost penalty of ultrahigh-purity atmosphere systems.

## Results

### Printability under trace-air-doping conditions

Pre-alloyed Ti_56_Zr_30_Nb_14_ (at.%) powders with uniform elemental distribution, high sphericity, and a single-phase BCC structure were chosen as the feedstock for additive manufacturing (Supplementary Fig. 1a–e). After additive manufacturing under trace-air-doping conditions, the surface vividly turned iridescent from atmospheric reaction (Supplementary Fig. 1f), yet industrial CT confirmed a virtually pore-free interior (>99.99% relative density) and absence of cracks (Supplementary Fig. 1g), evidencing robust printability even under trace-air-doping conditions. The normally distributed 25.9 µm pores observed here represent a typical porosity profile common to AM alloys (Supplementary Fig. 1h).

### Mechanical properties

**Fig. 1:**
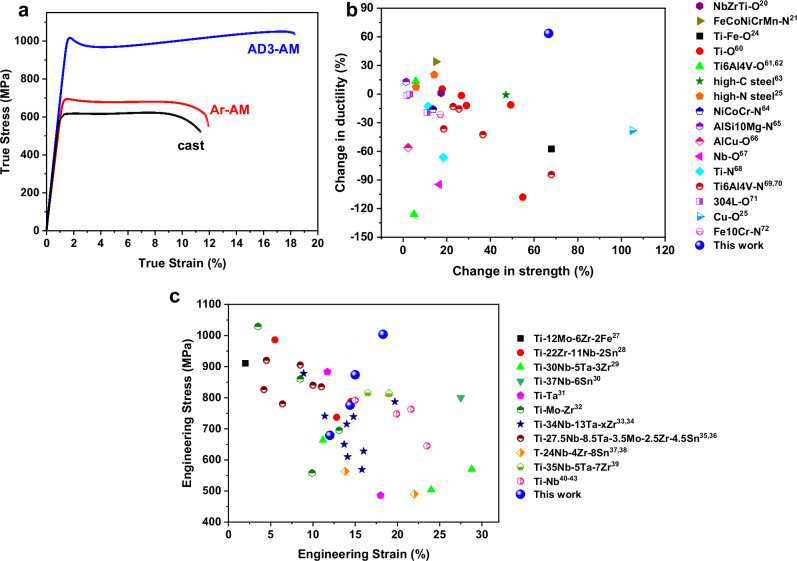
Tensile properties of the AD-AM alloy and comparison with other materials. **a** Representative stress-strain curves for the MEAs processed under different conditions (Ar-AM: high-purity Ar atmosphere; AD3-AM: air doping at gradient level 3). The optimally air-doped sample (AD3-AM) exhibits a simultaneous and significant improvement, with yield strength surpassing 1 GPa and elongation reaching ~18%, defying the conventional strength-ductility trade-off. **b** Comparison of strength increment against ductility change of the AD3-AM MEA with a broad set of AM alloys whose microstructures contain impurity elements (O, N, C) introduced either deliberately or inadvertently during manufacturing^20,21,24–26,60–72^. The AD3-AM alloy resides in the upper-right quadrant, representing a rare synergy of simultaneous strength and ductility enhancement. **c** Comparison of strength-ductility in AD-AM MEAs versus most reported AM low-modulus β titanium alloys^27–43^. The AD3-AM alloy demonstrates a superior combination of high strength and good ductility, positioning it as a promising candidate for load-bearing orthopedic implants.

As the air-doping level in the build chamber progressively increases, AM samples absorb more O and N, and their hardness increases accordingly (Supplementary Fig. 2a, b). This demonstrates that the introduced O and N atoms are fully dissolved into the alloy matrix during the AM process (Supplementary Note 1, Supplementary Fig. 3). Figure 1a compares stress-strain curves of the as-cast alloy with those produced under Ar-AM and air-doped AM (AD3-AM). The as-cast alloy yields at 600 MPa with 11% tensile elongation, whereas the Ar-AM specimen yields at 686 MPa and retains 12% elongation. The higher strength of the as-printed (Ar-AM) alloy over its cast counterpart stems from solid-solution strengthening by unintentionally incorporated interstitials: Ar-AM contains 0.089 wt% O and 0.030 wt% N, versus 0.030 wt% O and 0.004 wt% N in the as-cast material. Increasing the air-doping level to AD3 boosts the strength above 1 GPa while extending elongation to 18%. Ar-AM alloy suffers pronounced strain localization, triggering rapid necking right after yielding. Conversely, AD3-AM counterparts display markedly higher work-hardening capacity, suppressing necking and promoting uniform strain distributions (Supplementary Note 2, Supplementary Fig. 4).

Supplementary Fig. 2c clearly shows this trend: With the increasing introduction of air into the build chamber, both strength and plasticity rise concurrently, confirming that O/N impurity atoms absorbed from the atmosphere act as effective in situ alloying elements that provide potent solid-solution strengthening. Nonetheless, when the air-doping level is further increased (i.e., AD4), the sample underwent brittle fracture before macroscopic yielding, a reversal that may attributable to excessive O/N segregation at grain boundaries, as directly evidenced by APT (see Supplementary Fig. 5). It should be emphasized that the alloy printed with TiO_2_ addition exhibits significantly inferior performance to its counterpart fabricated via the atmosphere-mediated strategy (see Supplementary Note 3 and Supplementary Figs. 6, and 7).

Figure 1b compares changes in the mechanical properties of the AD3-AM MEA against those of a broad spectrum of AM alloys whose microstructures contain O, N, or C introduced either deliberately or inadvertently. Our AD3-AM alloy exhibits an anomalous response: yield strength surges by 67% while elongations simultaneously rise by 64%—a synergy rarely observed. In most studies (Ti-Fe-O, Cu-O, etc.^24,25^), large strength gains are accompanied by pronounced ductility loss; even the exceptional FeCoNiCrMn-N or high-N steels^21,26^ deliver only modest concurrent improvements. Figure 1c presents a comparison of tensile yield strength versus ductility between our AD-AM alloys and AM low-modulus β-Ti biomedical alloys reported in literature^27–43^. The AD3-AM yields a compelling strength-ductility synergy, endowing the alloy with 1 GPa yield strength and 67 GPa elastic modulus (Supplementary Fig. 8). Metallic implants demand high strength, low modulus, and critically, outstanding corrosion resistance^4,5^. The AD3-AM MEA meets all three requirements: its breakdown potential reaches 8 V, far surpassing conventional Ti alloys, while remaining only marginally below that of its Ar-AM counterpart (Supplementary Figs. 9–11). All these aspects highlight clinical viability and the great potential of the AD-AM MEAs for load-bearing implants.

### Microstructural characterization

**Fig. 2:**
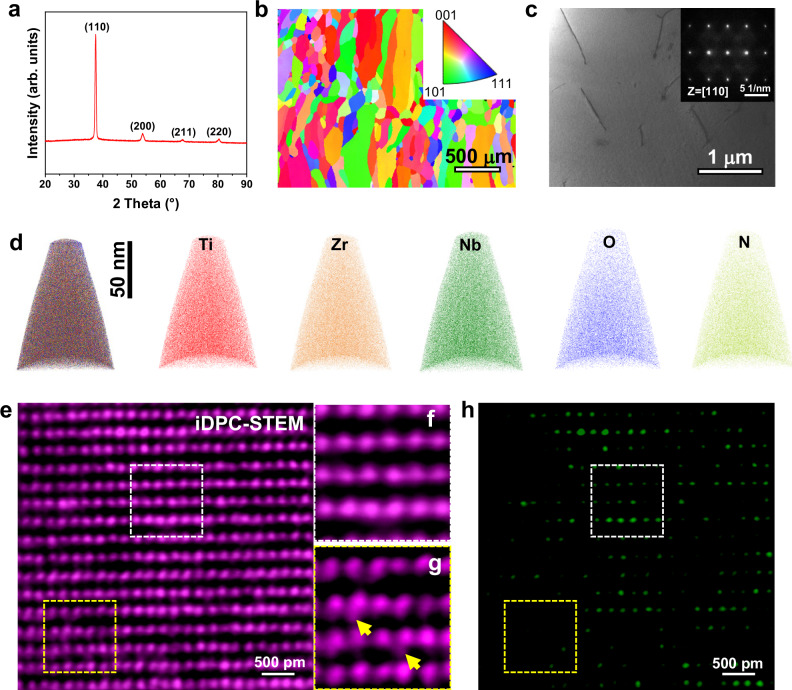
Microstructural characterization of the AD3-AM MEA. **a** XRD patterns confirming the alloy retains a single-phase BCC structure after printing, with no detectable oxide or nitride peaks, indicating all absorbed O and N are dissolved interstitially. **b** EBSD IPF images along the deposition direction. **c** Bright-field TEM image and the corresponding SAED pattern confirming the absence of second-phase precipitates. **d** APT elemental maps showing uniform distribution of Ti, Zr, Nb, O, and N at the sub-nanometer scale, with no evidence of clustering. **e** Integrated differential phase contrast (iDPC)-STEM image providing atomic-scale resolution. **f** Magnified view of the white-boxed region in (**e**). **g** High-magnification view of the yellow-boxed region in (**e**), with yellow arrows directly marking the atomically resolved interstitial O/N atoms within the BCC lattice. **h** Contrast-adjusted view of (**e**), revealing the preferential segregation of interstitial atoms to Ti/Zr-rich regions (darker contrast in the image).

Regardless of the air-doping level, AD-AM alloys retain a single-phase BCC lattice (Fig. 2a and Supplementary Fig. 12); no oxides or nitrides precipitate, confirming that all absorbed O and N dissolve interstitially as in situ alloying elements. Supplementary Fig. 13 provides the inverse pole figure (IPF) map of the as-cast alloy and the grain-size distribution histograms for all three key samples. In contrast to the equiaxed microstructure of the as-cast condition, the AM samples exhibit a discernible but limited difference in grain morphology. IPF images reveal that the Ar-AM alloy exhibits only limited epitaxial growth, with an average grain size of 112.5 µm (Supplementary Fig. 14a). The high melting point of the alloy accelerates solidification (large cooling rate of *R*), while its low thermal conductivity decreases the thermal gradient (small *G*); Consequently, the resulting low *G*/*R* ratio suppresses extensive epitaxial growth^44^. Although minor epitaxial features persist along the deposition direction, their length and aspect ratio remain substantially constrained. The AD3-AM counterpart displays almost identical microstructural features (Fig. 2b). Supplementary Fig. 14b presents a STEM-HAADF image of the Ar-AM sample, with the upper-right inset revealing a single-phase BCC matrix. In this image, brighter atomic columns correspond to heavier elements, revealing nanoscale chemical inhomogeneity^19^. Intensity line profiles across the red-boxed atomic columns corroborate this inherent compositional fluctuation at the atomic scale (Supplementary Fig. 14c).

Figure 2c presents a bright-field TEM image and the corresponding SAED pattern of the AD3-AM sample. Despite the substantial O/N uptake, the diffraction data reveal no nanoscale oxides or nitrides, confirming retention of a single-phase BCC lattice. It should be noted that the O and N atoms introduced during AM are uniformly dissolved in the matrix, with no detectable segregation at grain boundaries or melt-pool surfaces, and no formation of oxides or nitrides (Supplementary Figs. 15 and 16). Also, only a few sparse dislocation lines are visible with no dislocation tangles or cellular networks, a consequence of the reduced residual stresses afforded by the large beam diameter and slower cooling inherent to L-DED. Three-dimensional atom-probe tomography (3D-APT) further reveals uniform distributions of Ti, Zr, Nb, O and N at sub-nanometer scale; no solute clusters, precipitates or compositional fluctuations were detected (Fig. 2d). Figure 2e shows an integrated differential phase contrast (iDPC) image where individual interstitial atoms are directly resolved, as highlighted by yellow arrows in Fig. 2g. The contrast-adjusted STEM image in Fig. 2h reveals that these yellow regions are enriched in light atoms (Ti, Zr), whereas the white regions correspond to heavy, Nb-rich domains devoid of interstitials (Fig. 2f). This contrast pattern indicates that interstitial O/N atoms preferentially associate with Ti/Zr, forming potential ordered interstitial complexes (OICs)^18,21^.

### Machine-learning-enhanced atom-probe tomography

When oxygen is the only interstitial, iso-concentration analysis—as demonstrated by Lei et al.—suffices to reveal Ti-Zr-O ordered complexes that can be validated by aberration-corrected ABF-STEM^18^. In the present AD3-AM MEAs, deliberate co-doping of O and N yields intertwined interstitial clusters whose internal chemistry evades both iDPC/ABF-STEM (because O and N exhibit overlapping signals and negligible Z-contrast) and conventional APT, whose lateral resolution (~0.3 nm) matches the BCC octahedral spacing^45^. Machine-learning-assisted analysis is therefore essential to resolve individual interstitial species and to quantify the full spectrum of clusters that underpin the abnormal strengthening in AD-AM MEAs.

**Fig. 3:**
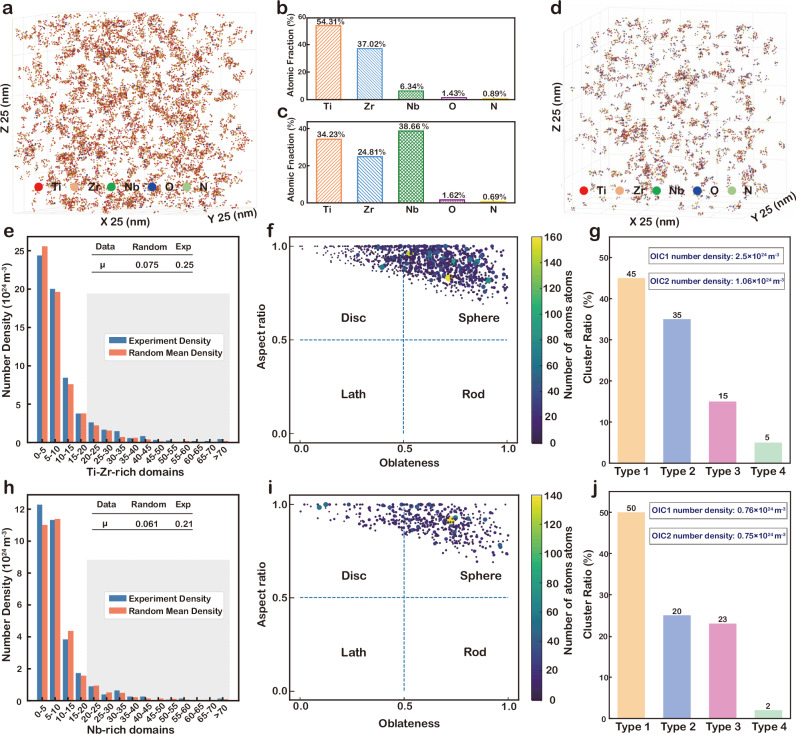
ML-APT characterization of OICs in AD3-AM alloy. **a**, **d** APT three-dimensional reconstructions of Ti-Zr-rich domains and Nb-rich domains from the same dataset, with different elements colored differently. **b**, **c** Elemental distribution maps within Ti-Zr-rich domains and Nb-rich domains, respectively, showing significant differences in chemical composition between the two types of domains. **e**, **h** Size distribution histograms of Ti-Zr-rich domains and Nb-rich domains, respectively, compared with the distributions from a chemically randomized dataset (“Methods” section and Supporting Information). The contingency coefficient (μ) between the experimental and randomized datasets is indicated. OIC sizes are expressed as the number of atoms counted by APT (ideally, 20 atoms correspond to a 0.5 nm^3^ cube). To clearly show the distribution trend, domains containing more than 70 atoms are merged into a single bin. **f**, **i** Morphology maps of Ti-Zr-rich domains and Nb-rich domains, respectively. The size and color of each circle represent the number of atoms within a single domain (larger circles and yellower hues indicate larger domains containing more atoms). **g**, **j** Proportions of different types of OICs (types 1–4) within Ti-Zr-rich domains and Nb-rich domains, respectively. The number densities of type 2 and type 3 (i.e., OIC1 and OIC2), which play major strengthening roles, are also indicated in the figures.

To overcome the challenge of resolving typical OICs in APT data, we developed an ML-APT workflow by optimizing the approach of Li et al.^45^ for enhancing the detection of OICs. (See “Methods” section, Supplementary Note 4 and Supplementary Figs. 17 and 18 for details). Figure 3a, d shows the 3D distribution of Ti-Zr-rich and Nb-rich domains identified by ML-APT, including pristine SROs and clusters alongside O/N-interstitial-reinforced counterparts (i.e., OICs), the latter being further classified into two distinct chemical families. The corresponding elemental distributions are presented in Fig. 3b, c, consistent with previous reports^19,46^. Size distributions of the domains are given in Fig. 3e, h. We employed the normalized contingency coefficient μ (0 = random, 1 = maximal deviation) to verify statistically significant, non-random clustering of Ti-Zr-rich and Nb-rich domains^47^; an experimental μ exceeding the random benchmark confirms non-randomness. Considering that the average SRO diameter is about 0.7 nm^48^, clusters containing fewer than 20 atoms are mostly disordered. Hence, subsequent analyses are restricted to domains with >20 atoms (gray-shaded region). The number density of Ti-Zr-rich domains is approximately five times that of Nb-rich domains, attributed to the stronger Ti-Zr bonding tendency and the higher fraction of Ti/Zr in the alloy^19^. Typical spatial distributions of the Ti-Zr-rich and Nb-rich domains are shown in Fig. 3f, i, which exhibit near-spherical morphologies. As shown in Fig. 3g, j, the Ti-Zr-rich and Nb-rich domains can be categorized into four types: types 1–3 (type 1: without interstitial elements; type 2: O-containing interstitial strengthening, defined as OIC1; type 3: N-containing interstitial strengthening, defined as OIC2), and type 4 (containing both O and N). It is worth noting that the type 4 variant appeared only sporadically and is therefore disregarded here. Ti-Zr-rich and Nb-rich domains remain predominantly free of interstitials, indicating that not all pristine SROs or clusters are supplanted by OICs. Quantitatively, within Ti-Zr-rich domains, OIC1 (Type 2: O-Zr-Ti complexes) exhibits a number density of 2.5 × 10^24^ m^−3^, exceeding that of OIC2 (Type 3: N-Zr-Ti) at 1.06 × 10^24^ m^−3^, together, they constitute the two dominant OIC populations in this system. Nb-rich domains host only sparse O- and N-bearing OICs, with number densities markedly lower than those in Ti-Zr-rich domains.

## Discussion

Based on ML-APT analysis and mechanical modeling, a detailed quantitative discussion follows to clarify the individual contributions to the reinforcement. In the AD3-AM alloy, the increase in yield strength stems from the O/N interstitials (2.24 at.%), whose lattice distortion strongly interacts with dislocations^18,49^. Consequently, the classical interstitial solid-solution strengthening model is employed to quantify the contribution of O/N atoms, as shown in Eq. 1^19^:

1 $$\varDelta \tau=\frac{G\varDelta \epsilon {c}^{1/2}}{3}$$

Where *G* is the shear modulus (taken as 23.7 GPa according to [19]), *Δϵ* represents the lattice mismatch parameter corresponding to the distortion induced by interstitial atoms (with values of 0.1 for O and 0.14 for N, respectively^17^), and *c* denotes the atomic concentration of interstitial atoms in the solid solution. Classical solid-solution modeling attributes ~257 MPa to these atoms, of which randomly dissolved interstitials (~90% of the total) contribute ~231 MPa.

While approximately 90% of the randomly distributed interstitial atoms constitute the fundamental basis for alloy strengthening, the remaining ~10% incorporated into OICs serve as the critical catalyst for achieving a significant leap in mechanical performance. These OICs exhibit distinctly different contribution logics dictated by the chemical characteristics of their central atomic domains. By considering the two primary chemical environments (Ti-Zr-rich and Nb-rich domains), four sub-types of OICs are identified and quantified. Type 2 (OIC1) accounts for approximately 6.5% of the total interstitials: the Ti-Zr domains OIC1, possessing the highest occupancy (~4.2%), contribute more to strengthening compared with the smaller Nb-Nb domains OIC1 (~2.3%). Crucially, these specific structures effectively promote dislocation cross-slip and enhance dislocation storage capacity^18^, and thus contribute to the high ductility^21^. In contrast, type 3 (OIC2) encompasses approximately 3.5% of the interstitials, where the Ti-Zr domains (~2.1%) and the Nb-Nb domains (~1.4%) fractions contribute a moderate strength increment and supplementary reinforcement, respectively. This synergistic combination further increases the alloy’s strength without sacrificing its ductility^21,50^.

Therefore, the reinforcement mechanism emerges from a synergistic interplay of four domain types: the randomly dissolved interstitials (~90%) provide the solid-solution strengthening; the ordered OICs (Types 2 & 3, ~10%) act as potent, correlated obstacles that deliver additional strengthening and ductility enhancement; the pure metal domains (Type 1) form the inert matrix; and the negligible Type 4 domains have minimal influence. In-depth analysis from physical modeling reveals that classical point-obstacle models predict a total theoretical strength lower than experimental values; this discrepancy precisely illuminates the strengthening essence of OICs as finite-sized obstacles. Distinct from the isolated solute point obstacles in traditional models, OICs—with a radius of approximately 0.7 nm—possess significant internal order, which induces a more potent synergistic stress field within the lattice^51,52^.

Furthermore, in the Ti-Zr-rich domains, the number densities of OIC1 (O-Zr-Ti) and OIC2 (N-Zr-Ti) evolve systematically with increasing air-doping level. At lower doping stages (AD1 and AD2), their densities remain relatively low. When the doping level increases from AD2 to AD3, the OIC1 number density more than doubles—from 1.19 × 10^24^ m^−3^ to 2.50 × 10^24^ m^−3^—while OIC2 also shows a concurrent rise from 0.77 to 1.06 × 10^24^ m^−3^. However, in the excessively doped AD4 sample, both OIC1 and OIC2 densities decrease to 2.10 and 0.90 × 10^24^ m^−3^, respectively. In Nb-rich domains, OICs remain sparse at lower doping levels; at the AD3 stage, both OIC1 (O-Nb) and OIC2 (N-Nb) densities rise to 0.76 and 0.75 × 10^24^ m^−3^, and at AD4 the OIC1 density further increases to 0.90 × 10^24^ m^−3^ while OIC2 shows a decrease to 0.59 × 10^24^ m^−3^, yet they remain substantially lower than those in the Ti-Zr-rich domains (Supplementary Fig. 19).

Correspondingly, the mechanical properties improve systematically up to the AD3 doping level. As shown in Supplementary Fig. 2, with the increase in total O/N content from 0.268 wt.% (AD1-AM) to 0.510 wt.% (AD3-AM), the yield strength rises from 776 MPa to 1002 MPa, the elongation improves from 14% to 18%, and the hardness increases from 254.6 HV to 343.1 HV. By intensifying lattice distortion (for solid-solution strengthening) and promoting the formation of intragranular OICs, elevated interstitial content thus underpins the observed strength-ductility synergy. However, at the highest doping level (AD4-AM), mechanical performance deteriorates due to pronounced segregation of excess interstitials at grain boundaries (Supplementary Fig. 5). This leads to embrittlement rather than further beneficial OIC formation, accounting for the plateau in hardness (346.5 HV) and the loss of ductility. The straightforward control of microstructure and resulting properties, achieved simply by adjusting the air-doping level in the build chamber, demonstrates exceptional tunability.

To elucidate the origin of the exceptional ductility and work-hardening ability in AD3-AM samples, deformation microstructure was tracked before and after deformation. Pre- and post-deformation TEM microstructures (Supplementary Fig. 20) and XRD patterns (Supplementary Fig. 21a) confirm that all the samples retain a single-phase BCC lattice, ruling out transformation-induced plasticity (TRIP) as a work-hardening source. No deformation twins were observed (Supplementary Fig. 21b, c); instead, plasticity proceeds via kink bands with ~2° misorientation that evolve into low-angle grain boundaries, rerouting slip and sustaining hardening when TRIP or TWIP is inactive^53^. Post-deformation EBSD reveals more uniform strain distribution in the AD3-AM samples than in the Ar-AM samples, and fracture surfaces exhibit finer, denser dimples (Supplementary Fig. 22), consistent with digital image correlation results (Supplementary Fig. 4).

**Fig. 4:**
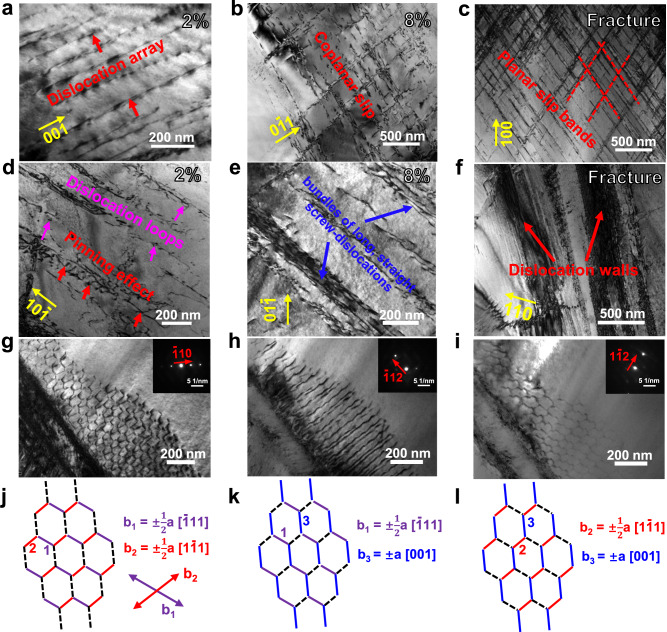
Deformation microstructure along the [110] zone axis in pre-strained and fractured Ar-AM and AD3-AM samples. **a**–**c** Bright-field TEM images of Ar-AM samples deformed to 2% strain, 8% strain, and after fracture, respectively, showing relatively planar dislocation slip. **d**–**f** Corresponding bright-field TEM images of the AD3-AM alloy at matched deformation strains, revealing dislocation behavior regulated by nanoscale OICs: **d** At 2% strain, distinct OIC-induced dislocation pinning and curved dislocation loops are observed, indicating the early activation of cross-slip; **e** At 8% strain, long, straight bundles of screw dislocations are formed; **f** After fracture, well-developed dislocation walls are visible, confirming sustained cross-slip of screw dislocations during the entire plastic deformation process. **g**–**i** Two-beam bright-field TEM images of dislocation networks in the AD3-AM sample pre-strained to 8%, imaged under different diffraction vectors. **j**–**l** Hexagonal arrays showing the reaction of two glissile 1/2〈111〉 dislocations into an immobile 〈100〉 segment, contributing to sustained work hardening.

Figure 4a–c shows the evolution of dislocation morphology in Ar-AM samples. Planar slip dominates the entire plastic deformation process, as evidenced by planar slip bands observed. Once slip initiates on favored planes, successive dislocations pile up rapidly on the same planes, concentrating stress and extinguishing work-hardening capacity. In stark contrast, AD3-AM specimens (Fig. 4d–f) show immediate deviation from planar slip. At 2% strain, curved dislocation loops (pink arrows) and discrete pinning points (red arrows) emerge to reveal active cross-slip triggered by OICs acting as effective dislocation obstacles. At 8% strain, long and straight screw-dislocation bundles emerge. After fracture, intertwined dislocation walls display pronounced wavy slip and frequent cross-slip, evidencing sustained activation of cross-slip throughout deformation. OICs structures facilitate plastic deformation via dual mechanisms: (i) they pin dislocations yet provide paths for dislocations to bypass obstacles through cross-slip, averting stress concentration; and (ii) by generating fresh dislocations and point defects via pin-and-cut processes, which enhances dislocation multiplication capacity^21,23^. This synergistic effect yields exceptional strength-ductility balance in OICs-strengthened alloys, underscoring multi-scale dislocation interactions throughout the deformation process.

Figure 4g–i captures dislocation networks in the AD3-AM specimen pre-strained to 8%, where hexagonal networks display distinct configurations under varying g-vectors. It is well established that dislocation glide preferentially proceeds along close-packed planes and directions to minimize the system energy in accordance with the principle of energy minimization^54^. Experimental and computational investigations have demonstrated that most mobile dislocations in BCC metals bear a Burgers vector of 1/2<111>, where <111> represents the closely packed direction^55–58^. During plastic deformation, extensive glide fosters frequent dislocation interactions and rearrangements that effectively redistribute strain and sustain work hardening. Specifically, two glissile 1/2<111> dislocations react to form an immobile <001> dislocation (a/2<1$\bar{1}$1>+a/2<$\bar{1}$11> → a<001>), as shown in Fig. 4j–l, generating subgrain-boundary dislocation arrays. These arrays relieve boundary stress and operate as Frank-Read sources for dislocation multiplication. Typically, they constitute incipient low-angle grain boundaries that provide slip transfer paths for dislocations to traverse grain boundaries. These configurations effectively alleviate stress concentration and dislocation pile-ups at boundary regions^59^.

**Fig. 5:**
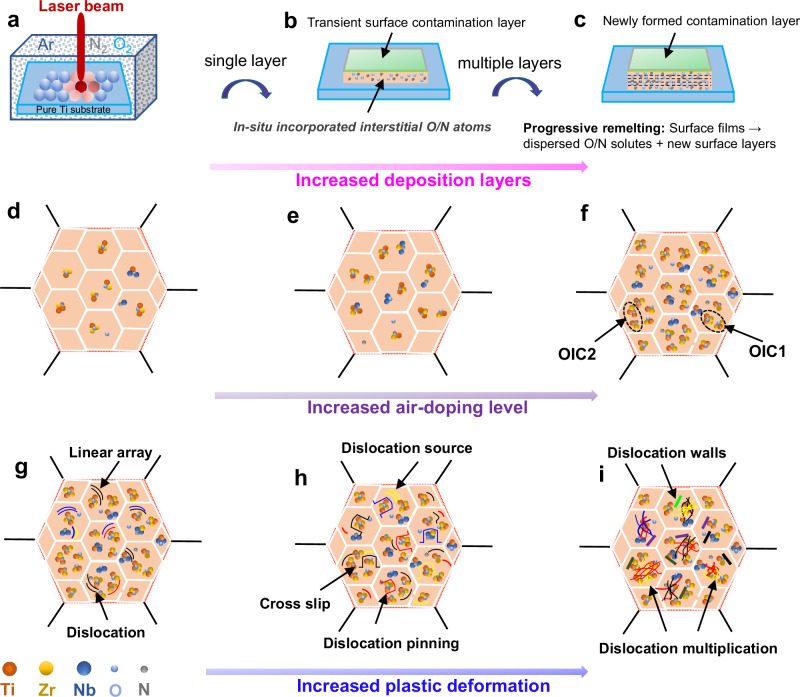
Schematic illustration of in situ O/N alloying, OICs evolution, and deformation mechanisms. **a**–**c** Step-by-step depiction of the atmosphere-mediated in situ alloying process during AM: **a** Atmospheric O/N species react with the high-temperature melt pool during layer-wise printing; **b** Gaseous O/N dissolve into the solidifying printed layer while a transient oxide/nitride surface film forms; **c** Progressive remelting induced by subsequent printed layers breaks down the transient surface film, and cyclic in situ reheating during the AM drives uniform homogenization of O/N solutes throughout the printed matrix. **d**–**f** Correlation between air-doping level and OIC formation: OIC number density in the as-deposited samples rises markedly with increasing air-doping level, revealing the structural origin of the enhanced mechanical performance in the AD-AM MEA relative to the cast and Ar-AM control groups. **g**–**i** Deformation mechanism in the AD-AM MEA: during plastic straining, the homogenously dispersed OICs effectively pin mobile dislocations, trigger frequent cross-slip events, and act as stable Frank-Read sources, which simultaneously enhances the work-hardening capacity and preserves tensile ductility.

Our AD-AM route protocol turns atmospheric O and N into deliberate alloying elements. Each melt pass first scavenges introduced N_2_/O_2_ (Fig. 5a), then during rapid solidification, these atoms solid solutionized into the matrix while a transient film forms on the surface (Fig. 5b). During subsequent passes, the prior surface film remelts under cyclic heating, re-alloying into the matrix, homogenizing the interstitials before a fresh layer again reacts with the atmosphere (Fig. 5c). Within the deposited alloy, increasing the air-doping level raises the amount of introduced O/N, and repeated thermal cycles nucleate two distinct OIC structures (Fig. 5d–f). The introduction of increased OICs during AD-AM significantly modulates dislocation behavior and deformation mechanisms in subsequent plastic deformation. During initial straining, linear dislocation arrays form, where dislocation glide is pinned by OICs (Fig. 5g). Progressive straining triggers extensive cross-slip, which alleviates stress concentration during plastic deformation. Moreover, OICs act as Frank-Read sources that continuously emit dislocations, enhancing dislocation multiplication and elevating work-hardening capability (Fig. 5h, i).

In summary, we introduced an atmosphere-mediated impurity-engineering strategy that exploits in situ available O and N during AD-AM to tailor microstructure and mechanical properties of TiZrNb MEAs. This yields an unprecedented strengthening protocol: 67% gain in yield strength (≈1 GPa) alongside 64% higher ductility (≈18%). Our analysis reveals two tunable interstitial-strengthening motifs: O-rich OIC1 and N-rich OIC2. These OIC motifs can effectively pin dislocations yet trigger prolific dislocations, cross-slip, and Frank-Read multiplication, sustaining work hardening without embrittlement. Such atmosphere-tuned O/N alloying during AM breaks the classic strength-ductility trade-off and provides a scalable, cost-effective route to high-performance biomedical implants.

## Methods

### Materials fabrication

Ti-Zr-Nb MEA powders, supplied by Guangzhou Sailong Additive Manufacturing Co., Ltd. (China), were fabricated via electrode induction melting gas atomization (EIGA). The powder exhibited a particle size distribution of 40–105 µm, with a nominal composition of Ti_56_Zr_30_Nb_14_ (at%). AM experiments were performed using a 6 kW YLS-6000 fiber laser additive manufacturing system (IPG Photonics, Germany) with coaxial powder feeding and a 1.5 mm focused beam diameter at the substrate surface. The MEA powders were delivered through four argon-purged coaxial nozzles at 0.8 rpm feed rate, achieving 50% track overlap for continuous multilayer deposition. Laser power of 800 W, a scan speed of 480 mm/min, and a nominal layer thickness of 0.4 mm were utilized, and final products with build dimensions of 52 mm (length) × 12 mm (width) × 10 mm (height) were produced.

A commercially pure titanium substrate was selected based on thermal expansion coefficient matching to minimize interfacial stresses. The initial printing was conducted under a high-purity argon shield, after which a precisely controlled flow of air was deliberately introduced into the argon-filled chamber. The printing was performed under five distinct atmosphere conditions, designated as Ar-AM, AD1-AM, AD2-AM, AD3-AM, and AD4-AM. These labels correspond to oxygen levels of approximately 100, 3.0 × 10^3^, 1.3 × 10^4^, 2.4 × 10^4^, and 3.0 × 10^4^ ppm, respectively, showcasing a deliberate gradient of in situ alloying agent content. During actual printing, the in-chamber O contents fluctuated and declined progressively; after each sample was printed, the sensor-indicated oxygen level had fallen by 1000–1500 ppm relative to the initial reading, presumably owing to absorption by the sample. The N content in the processing atmosphere was estimated by maintaining the ambient atmospheric N_2_/O_2_ ratio. The chamber O levels displayed herein correspond to direct measurements from real-time O sensor monitoring during deposition. O and N contents in all the AM samples were measured by a LECO instruments inert gas fusion (IGF) machine with IR detection.

### Microstructural characterization

Microstructural observations were performed using a field-emission scanning electron microscope (FE-SEM, Zeiss Supra55), and EBSD analyses were conducted using an Oxford Instruments NordlysNano electron backscatter diffraction (EBSD) detector. EBSD measurements were conducted at an accelerating voltage of 20 kV, a sample tilt angle of 70°, and a scanning step size of 5 μm, with optimized beam conditions to ensure high pattern quality. The EBSD data was analyzed using the AZtecCrystal software, with a final indexing rate ≥90%; only data points with a mean angular deviation (MAD) ≤ 1.0° were retained for subsequent analysis. Phase identification and structural characterization were performed by using a Rigaku X-ray diffractometer (XRD) with Cu Kα radiation in a scanning 2θ range of 20°–90° at a rate of 10°/min.

Three-dimensional defect analysis of the as-printed specimens was carried out using high-resolution micro-computed tomography (μ-CT, DIONDO D2) with a tungsten anode. X-ray source parameters were optimized at 110 kV and 100 mA. Dislocation substructures in deformed specimens were examined using a Tecnai G20 transmission electron microscope (TEM) under dual-beam conditions, whilst atomic-scale structural analysis of AM samples was carried out using an aberration-corrected Thermo Fisher Spectra 30. For the TEM observation, specimens were mechanically ground to 50 μm thickness, and then twin-jet electropolished using H_2_SO_4_ (10%) and CH_4_O (90%) solution under −30 °C.

APT experiments were carried out in a local electrode atom-probe system (CAMECA Instruments LEAP 5000XR). The specimens were analyzed in voltage mode under an ultrahigh vacuum of approximately 2.5 × 10^−11^ Torr at 70 K, a target evaporation rate of 3 ions for 1000 pulses on average. The APT specimens were prepared by using Helios G5 UXFEI focused-ion beam (FIB)/SEM. The CAMECA integrated visualization and analysis software, IVAS 3.8.0, was used for data processing and three-dimensional (3D) atomic reconstruction.

### Mechanical testing

Microhardness was measured with a 7MHVS-50A tester at 1000 g load, and 15 s dwell time. Eight indentations were tested per sample, with results presented as the mean value. Dog-bone-shaped tensile specimens with a gauge dimension of 20 mm (length) × 4 mm (width) × 1.5 mm (thickness) were cut from the AM samples perpendicular to the deposition direction using electrical discharge machining. Tensile tests were conducted on a CMT4105 universal electronic tensile machine at room temperature under a strain rate of 5 × 10^−4^ s^−1^. Prior to deformation, the samples were polished down to a 2000-grit SiC paper. Each sample condition was repeated three times. A set of Digital images correlated (DIC) system was employed along with it for the in situ strain field analysis (Vic-2D, Correlated Solution).

### Corrosion performance testing

Electrochemical specimens (10 mm × 10 mm × 3 mm) with an exposed area of 1 cm^2^ were cleaned with acetone, connected with copper wires via platinum welding, cold-mounted, and ground to 2000 grit. The electrolyte was a 9 g·L^−1^ NaCl solution, deaerated by N_2_ bubbling for 4 h prior to testing. A standard three-electrode cell was employed: a platinum sheet counter electrode facing the working electrode, and an SCE reference electrode positioned 2–3 mm above the specimen surface. Potentiodynamic polarization was conducted from −0.3 V vs. OCP toward the anodic direction at 1 mV·s^−1^.

### ML-APT framework

To tackle the persistent challenge of accurately identifying SROs and OICs in APT datasets, we present ML-APT, a machine-learning-driven framework. Compared with conventional SDM-based modeling approaches^47^, this method achieves systematic identification and validation of SROs and OICs in APT data by introducing physical priors and a hierarchical recognition strategy. Specifically, the framework first exploits the difference in SRO values of Ti-Zr, Zr-Ti, and Nb-Nb pairs relative to the BCC matrix as prior features to construct an SRO recognition model. To this end, we established a crystal structure database containing both BCC and various SRO structures, from which the SRO values of the target element pairs were extracted. These SRO values were then used as inputs, with the corresponding crystal structure types as labels, to build training, validation, and testing datasets. A random forest algorithm was subsequently employed to train the recognition model (see Supplementary Note 3: Workflow of ML-APT for details of the training procedure). On this basis, a nearest-neighbor search strategy was further integrated, enabling the incorporation of adjacent O/N atoms into the identified SRO units and thus allowing the recognition of both SROs and potential OICs. Through comparative tests against randomized data, ML-APT can effectively distinguish genuine SROs and OICs from statistical noise, while simultaneously quantifying their number density and morphology.

### Reporting summary

Further information on research design is available in the Nature Portfolio Reporting Summary linked to this article.

## Supplementary information

Supplementary Information

Reporting Summary

Transparent Peer Review file

## Source data

Source Data

## Data Availability

The data that support the findings of this study are available from the corresponding author upon request. Source data are provided with this paper.

## Code availability

The custom code used in this study is publicly available on GitHub at https://github.com/838615495/ML-APT-OCS.
