# Supplementary material for: Strength-ductility synergy in medium-entropy alloys via harnessing trace air in additive manufacturing
Source: Nat Commun. 2026 Apr 29;17:5870. doi: 10.1038/s41467-026-72511-8 (PMC13333971; doi:10.1038/s41467-026-72511-8)
Supplement: Supplementary file 1 — Supplementary Information [file 41467_2026_72511_MOESM1_ESM.pdf]

Supplementary Information

Strength-Ductility Synergy in Medium-Entropy Alloys via Harnessing Trace Air in Additive Manufacturing

Yansheng Li[1†], Jiawei Yin[1†], Meiyuan Jiao[2], Tengfei Zheng[1], Yuan Wu[1,3]*, Shimiao Li[1], Guohui Zhang[1], Jiabin Yu[1], Yunzhuo Lu[4], Chun Shang[4], Haiou Yang[5], Yang He[6], Huihui Zhu[1], Sheng Zhang[7], Xiaobin Zhang[1], Xiongjun Liu[1], Suihe Jiang[1], Hui Wang[1], Zhaoping Lu[1,]*

[1]State Key Laboratory for Advanced Metals and Materials, University of Science and Technology Beijing, Beijing 100083, China
[2]Research Institute of Advanced Materials (Shenzhen) Co., LTD, China lron & Steel Research Institute Group, Shenzhen 518045, China
[3]School of Mathematics and Physics, University of Science and Technology Beijing, Beijing 100083, China
[4]School of Materials Science and Engineering, Dalian Jiaotong University, Dalian 116028, China
[5]State Key Laboratory of Solidification Processing, Northwestern Polytechnical University, Xi'an 710072, China
[6]Beijing Advanced Innovation Center for Materials Genome Engineering, School of Materials Science and Engineering, University of Science and Technology Beijing, Beijing 100083, China
[7]School of Mechanical Engineering, University of Science and Technology Beijing, Beijing 100083, China

[*] Corresponding at: wuyuan@ustb.edu.cn (Y. Wu); luzp@ustb.edu.cn (Z. Lu)
[†] These authors contributed equally to this work

This file contains the following supplementary materials:

Supplementary Figs. 1 to 22
Supplementary Note 1. In-situ incorporation of O/N impurities
Supplementary Note 2. Strain localization vs. homogeneity: DIC analysis of AM alloys
Supplementary Note 3. Mechanical and microstructural evaluation of oxide-doped AM TiZrNb alloys
Supplementary Note 4. ML-APT workflow for short-range order and ordered interstitial complexes
Supplementary references

Supplementary Notes

Supplementary Note 1. In-situ incorporation of O/N impurities

Deliberate air introduction during L-DED enables rapid incorporation of oxygen and nitrogen into the first deposited layer. Supplementary Fig. 3a shows SEM micrograph of the first layer in the AD3-AM sample. Supplementary Fig. 3b exhibits an enlarged view of the local area in (a), revealing a large number of nanoparticles. EDS analysis identified them as mixed oxides with minor nitrides (Supplementary Fig. 3c). These particles formed because the initially solidified surface underwent rapid oxidation/nitridation reactions in oxygen/nitrogen-enriched atmospheres during layer-by-layer deposition under air-doped conditions.

When additional layers were deposited, the native oxide/nitride film disappears. An SEM image acquired from the interior after multiple layers (Supplementary Fig. 3d) shows a featureless surface, and the corresponding EDS maps (Supplementary Fig. 3e) detected no particle-related O or N enrichment. This phenomenon is attributed to the inherent characteristics of L-DED: the larger laser spot diameter (≈1.5 mm) enables repeated re-melting of previously contaminated deposition surfaces, and the associated multiple thermal cycles dissociate the oxides/nitrides (Supplementary Fig. 3f). The liberated O and N atoms dissolved into the melt pool and were subsequently trapped in the solid as interstitial solutes, achieving a homogeneous distribution without discrete second-phase particles.

Supplementary Note 2. Strain localization vs. homogeneity: DIC analysis of AM alloys

To underscore the abnormal work-hardening response, full-field strain maps were captured via digital image correlation (DIC). The Ar-AM sample exhibits narrow strain localization bands oriented approximately 45° relative to the longitudinal axis at merely 2% plasticity, while minimal plastic strain was observed in other regions. At 8% plasticity, strain emanated from the centers of these bands, reaching a peak of 31.4% strain, accompanied by pronounced necking. Just before fracture, the entire deformation collapsed into a sub-millimeter zone, registering a catastrophic 68.5% local strain and leading to abrupt necking failure. Longitudinal strain profiles show localization by 8% plastic strain; as deformation further proceeded, the strain tightened into a 2 mm band along the gage length, marking severe localization just prior to fracture (Supplementary Fig. 4a).

Supplementary Fig. 4b illustrates strain evolution in the AD3-AM specimen. At 2% strain, a fragmented 45° band appeared, and by 8% strain the peak strain reaches only 16.6%—roughly half that of the Ar-AM sample. The strain field propagated steadily toward both grip ends, alleviating stress concentrations. Even at fracture, the peak strain was still limited to 36.2%. The corresponding axial strain profile shows that, at 8%, the AD3-AM specimen spread plastic strain over a markedly wider region than the Ar-AM counterpart. This spatially extended deformation suppressed necking and underpinned the superior uniform ductility of AD3-AM.

Supplementary Note 3. Mechanical and microstructural evaluation of oxide-doped AM TiZrNb alloys

To directly compare the two pathways, we introduced TiO_2 powder into the $Ti_{56}Zr_{30}Nb_{14}$ (at.%) matrix powder by ball milling at 100 rpm for 600 min under Ar atmosphere (Supplementary Fig.

6a). The printing parameters remained identical to those used for the AD-AM samples. After printing, TiO_2 peaks disappeared in the XRD pattern (Supplementary Fig. 6b), indicating decomposition during processing. At 1 at.% O, the yield strength exceeded 800 MPa but ductility remained low. At 2 at.% O, strength rose above 900 MPa, but ductility plummeted below 2%, with fractography revealing brittle intergranular fracture (Supplementary Fig. 6c-f). In contrast, the air-doped AD3-AM sample achieves a yield strength >1 GPa while maintaining >18% tensile ductility, and exhibits a ductile, dimpled fracture surface.

The underlying mechanism lies in microstructural homogeneity. In the oxide-doped sample, TEM reveals continuous Ti_2ZrO nanolayers (~10 nm thick) at grain boundaries (Supplementary Fig. 7), providing brittle pathways for crack propagation [1,2]. This phenomenon arises from the combination of rapid solidification and the intrinsic sluggish diffusion of the medium-entropy alloy. These impede the uniform distribution of oxygen, leading to its localized enrichment at grain boundaries where it kinetically favors the formation of continuous brittle nanolayers. Therefore, while both methods increase strength, the atmosphere-mediated approach uniquely avoids harmful grain-boundary phases, thereby preserving ductility and achieving a superior strength-ductility synergy.

Supplementary Note 4. ML-APT workflow for short-range order and ordered interstitial complexes

As shown in Supplementary Fig. 17, we propose a ML (machine learning)-APT (atom-probe tomography) method to identify typical element-rich domains and ordered interstitial complexes (OICs) in APT data. Using identification of Ti-Zr-rich domains as an example, the workflow consists of four main steps: data generation, APT detection, model training and parameter optimization, prediction and visualization.

Step 1: Data generation

In this study, we developed different compositional configurations through atomic simulations to construct a dataset for model development. Compared with the earlier work of Li et al. [3], the first improvement in our study lies in the choice of input features: instead of using spatial distribution map (SDM) curves, we employed scalarized chemical coordination preference parameters. This adjustment was made because, in the case of domains formed by elemental segregation, SDM curves fail to effectively capture the differences, whereas chemical coordination preference parameters can directly characterize the local chemical correlations between atoms.

Secondly, regarding the calculation of chemical coordination preference, we introduced a physics-informed Poisson-KNN statistical analysis method [4]. Originally applied to the APT data of GeSn alloys, this approach combines Kth-nearest neighbor (KNN) statistics with a Poisson distribution model to describe local chemical correlations, enabling three-dimensional mapping at the nanoscale. Compared with the traditional Warren-Cowley parameter, this method not only provides a more accurate description of local chemical segregation but also partially corrects biases and missing atoms inherent in APT detection. Based on this, we extended the method to the Ti-Zr-Nb alloy system, allowing more reliable analysis of chemical coordination preferences among different atomic pairs and thus revealing the formation characteristics of elemental enrichment domains. In particular, we focused on the Ti-Zr and Nb-Nb pairs as input features, since they

respectively represent heteroatomic affinity and homoatomic clustering, both of which are key to understanding the enrichment behavior of alloying elements.

On this basis, we constructed a BCC structure library, which includes (i) random solid solutions and (ii) two representative elemental enrichment domain modes (Ti-Zr-rich and Nb-rich). Each configuration was generated via atomic simulations, and its corresponding chemical coordination preference parameters were calculated using the improved method described above. Finally, the dataset consists of chemical coordination preferences as input features and structural categories (BCC matrix or specific enrichment modes) as labels, which were further divided into training, validation, and test subsets for model training, hyperparameter optimization, and performance evaluation.

Step 2: Simulated APT detection and voxelization

To reproduce experimental conditions and APT data characteristics, noise and detection efficiency effects were introduced into the simulated data (parameters detailed in [5]) to generate physically realistic training samples. After voxelization, the data were converted into a matrix format suitable for machine learning input, where each row represents a sample and each column corresponds to the SRO features of a specific atomic pair.

The voxelization workflow includes:

1. Data voxelization: converting atomic coordinates into a 3D voxel representation.
2. Adding noise and simulating detection efficiency: simulating experimental conditions by incorporating noise and adjusting detection efficiency.
3. Training sample construction: generating an $N \times 3$ matrix for model training, where N is the number of samples and 3 represents voxel coordinates/features.

Step 3: Model training and parameter optimization

The voxelised, noise-augmented dataset was used as input to train an SRO identification model based on the random forest algorithm. During training, hyperparameters including the number of decision trees, maximum depth, and minimum samples per split were optimized (tuning parameters), and the best-performing model was selected based on validation set performance (find the best model). After training, as shown in Supplementary Fig. 18, the receiver operating characteristic curve (ROC) was used to evaluate the performance of each model. ROC curve is a graphical tool used to evaluate the performance of classification models. It demonstrates the classification ability of the model at different thresholds by plotting the relationship between true case rate and false positive case rate. The larger the area under the ROC curve (AUC), the better the model performance. The random forest model exhibits the best performance and can be used for subsequent prediction.

Step 4: Prediction and visualization

After model training, we deployed it on experimental APT reconstructions. The experimental three-dimensional atomic coordinates were voxelized and feature-encoded following the procedure in Step 2 to ensure consistency in feature representation. The prediction results were visualized as voxel blocks, with different colors representing distinct phases or structural types (e.g., BCC matrix or Ti-Zr-rich domains).

For all voxels predicted as Ti-Zr-rich domains, we further applied unsupervised algorithms to analyze the surrounding atomic distribution in order to extract potential locally ordered atoms, and

incorporated neighboring O/N atoms using a nearest-neighbor search strategy. Specifically, taking the central atom of the identified Ti-Zr-rich domain as the core, atoms within a radius of $r = 0.25$-0.35 nm were examined, and all O/N atoms within this range were assigned to the corresponding Ti-Zr-rich domain unit, forming OICs. After the initial identification of OIC units, we introduced the contingency coefficient (μ) as a statistical metric to examine whether the spatial distribution of atoms within OIC units exhibits significant non-randomness. By comparing the experimental data with randomized counterparts, if the μ of the experimental data is significantly higher than that of the randomized dataset, it indicates that atoms within the Ti-Zr-rich domains exhibit a pronounced ordering preference.

In terms of OIC classification, we distinguished them according to the overall occupancy ratio of interstitial atoms: (i) if O atoms dominate the occupancy in a given OIC unit, it is classified as OIC1; (ii) if N atoms dominate, it is classified as OIC2. This classification process was carried out by statistically analyzing the probability distributions of O/N occupancy under different local environments.

In addition to structural identification, the ML-APT framework also provides quantitative analysis capabilities, such as the number density, size distribution, and chemical composition of typical Ti-Zr -rich domains and OICs. Since these characteristics are highly sensitive to alloy processing conditions, the model output can further serve as an alternative indicator for evaluating the effectiveness of processing routes. As shown in Supplementary Fig. 19, the AD3-AM process outperforms AD2-AM, evidenced by a higher number density of Ti-Zr -rich domains and an increased fraction of OICs.

Supplementary Figures

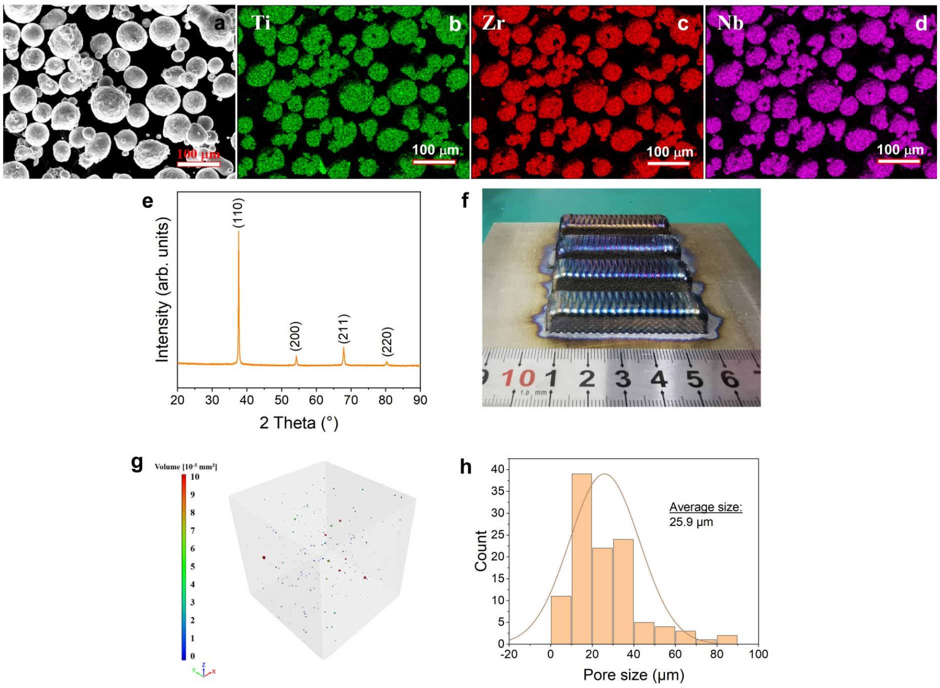

Supplementary Fig. 1 Characterization of feedstock and air-doped additively manufactured (AD-AM) materials. a, SEM morphology of the powders used. **b-d**, EDS elemental mapping of the powders. **e**, XRD pattern of the powder. **f**, Surface morphology of AD-AM samples. **g**, Industrial CT scan of a typical AD-AM sample. **h**, Pore distribution map of the AD3-AM sample.

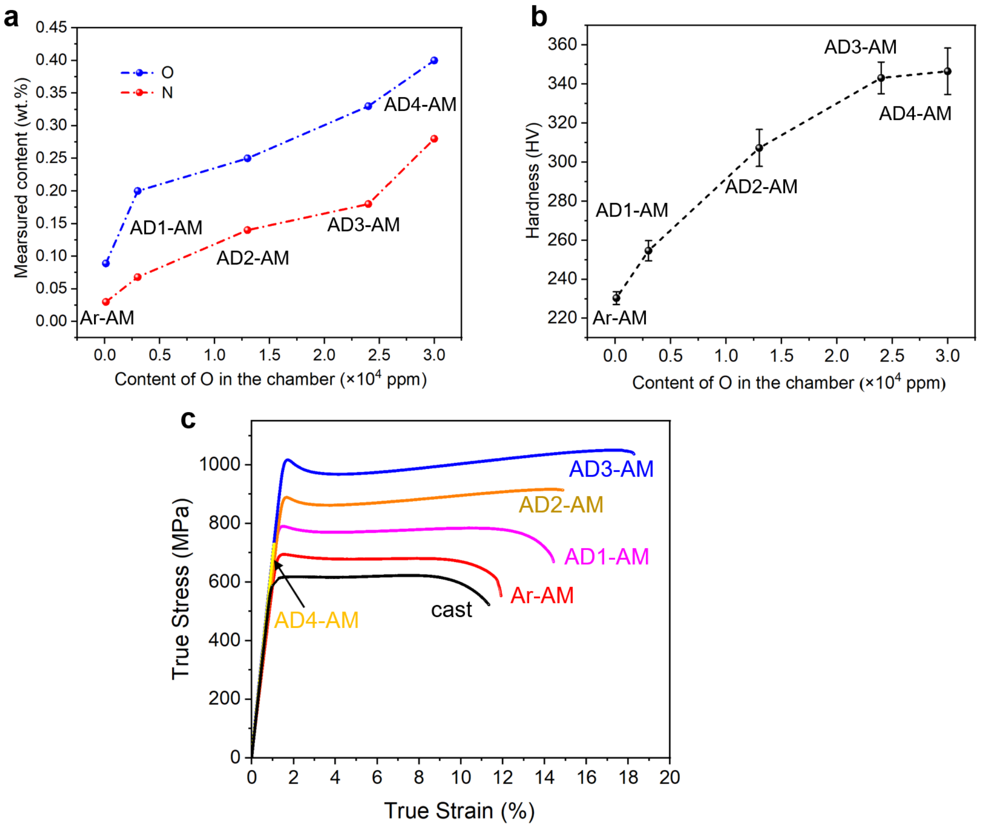

Supplementary Fig. 2 Influence of controlled gas environment on interstitial uptake and mechanical response. a, Measured O and N contents (wt.%) in AM samples fabricated under different atmosphere conditions (high-purity Ar-AM and gradient air doping groups AD1-AD4). **b**, Vickers microhardness of the AM samples with different air doping levels; error bars represent the standard deviation (SD) of at least 8 independent indentation tests. **c**, True stress-strain curves of the AM samples processed under Ar-AM and gradient air doping conditions.

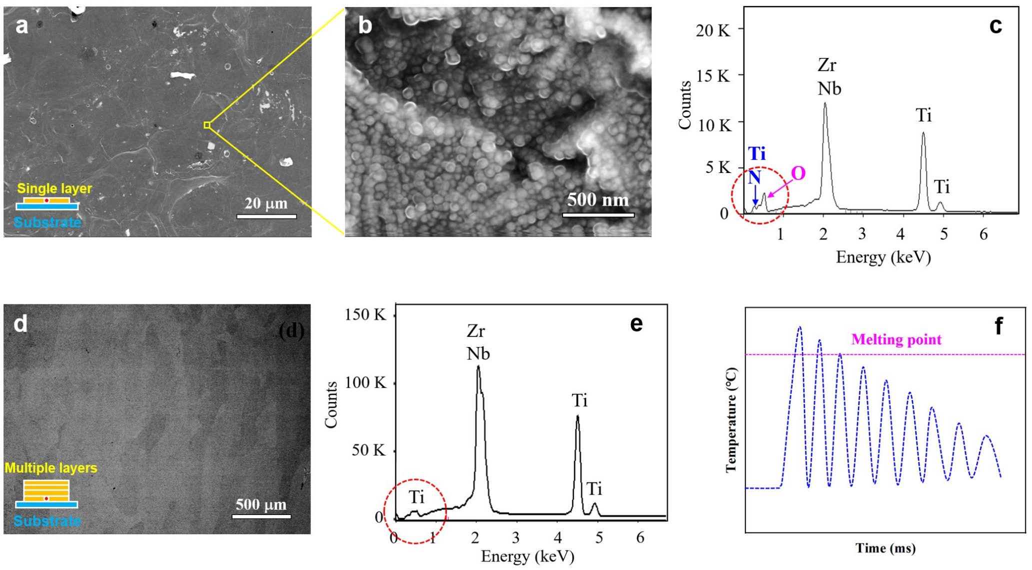

Supplementary Fig. 3 Characterization of surface contaminated layers after monolayer deposition versus interlayer microstructures in multilayer deposition under air-doping atmosphere. a, SEM image of the surface after single-layer deposition. **b**, High-magnification view of the boxed region in a. **c**, EDS spectrum acquired from the region shown in b. **d**, SEM image of an inner layer in a multilayer AM sample. **e**, EDS spectrum taken from the region shown in d. **f**, Schematic diagram of the thermal cycling experienced during multilayer deposition.

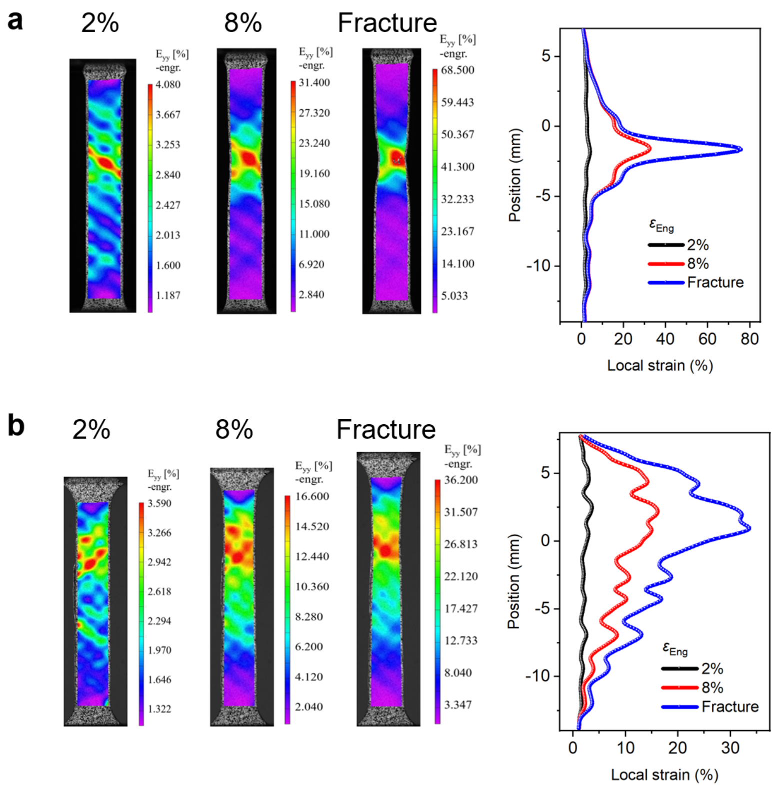

Supplementary Fig. 4 In-situ strain mapping of AM samples captured by digital image correlation (DIC). a and **b**, the local strain (ε_{yy}) distribution and the corresponding strain distribution profile for Ar-AM and AD3-AM samples deformed to 2% strain, 8% strain, and after fracture, respectively.

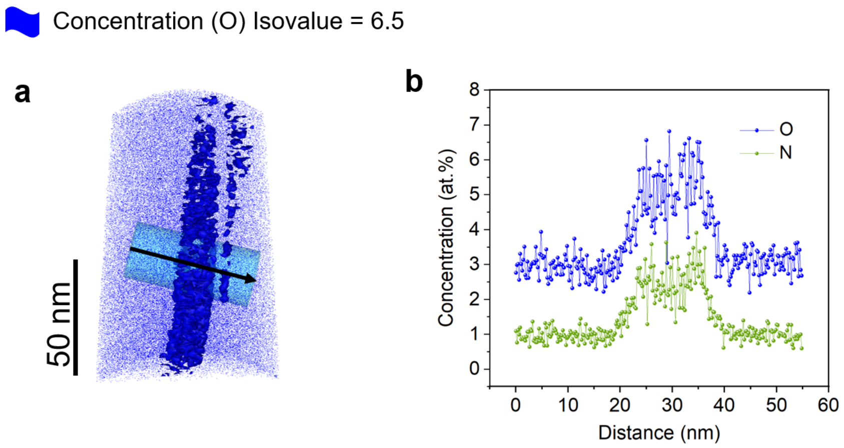

Supplementary Fig. 5 APT characterization of grain boundary segregation. a, 6.5 at.% oxygen isoconcentration surface of the APT specimen containing a grain boundary. **b**, 1D concentration profile along the black arrow in (a). Error bars correspond to the standard error (SE) of the concentration measurements.

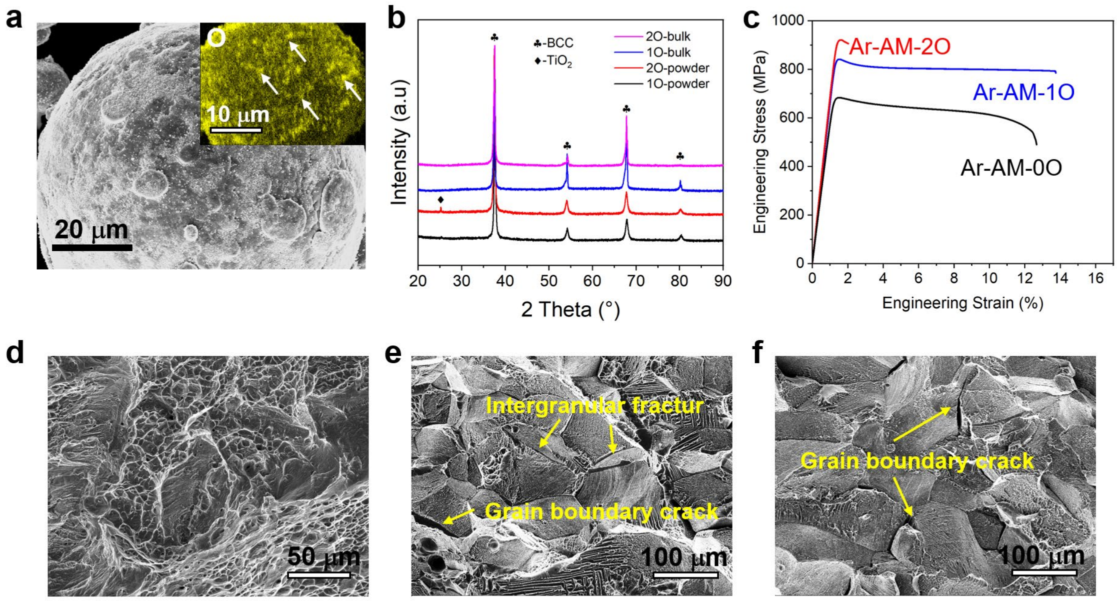

Supplementary Fig. 6 Effect of TiO_2-mediated oxygen doping on microstructure and mechanical properties of additively manufactured $Ti_{56}Zr_{30}Nb_{14}$ medium-entropy alloys. a, Morphology of the TiZrNb alloy powder after surface coating with TiO_2 powder (the inset shows the EDS mapping of O element; the particle indicated by the white arrow corresponds to TiO_2 powder). **b**, XRD patterns of the alloy powders doped with 1 at.% and 2 at.% O, and the XRD patterns of the corresponding alloys after additive manufacturing. **c**, Engineering stress-strain curves of the base alloy (Ar-AM-0O) fabricated by additive manufacturing, compared with those of the Ar-AM-1O and Ar-AM-2O alloys. **d-f**, Fracture surface morphologies of the Ar-AM-0O, Ar-AM-1O, and Ar-AM-2O alloys, respectively.

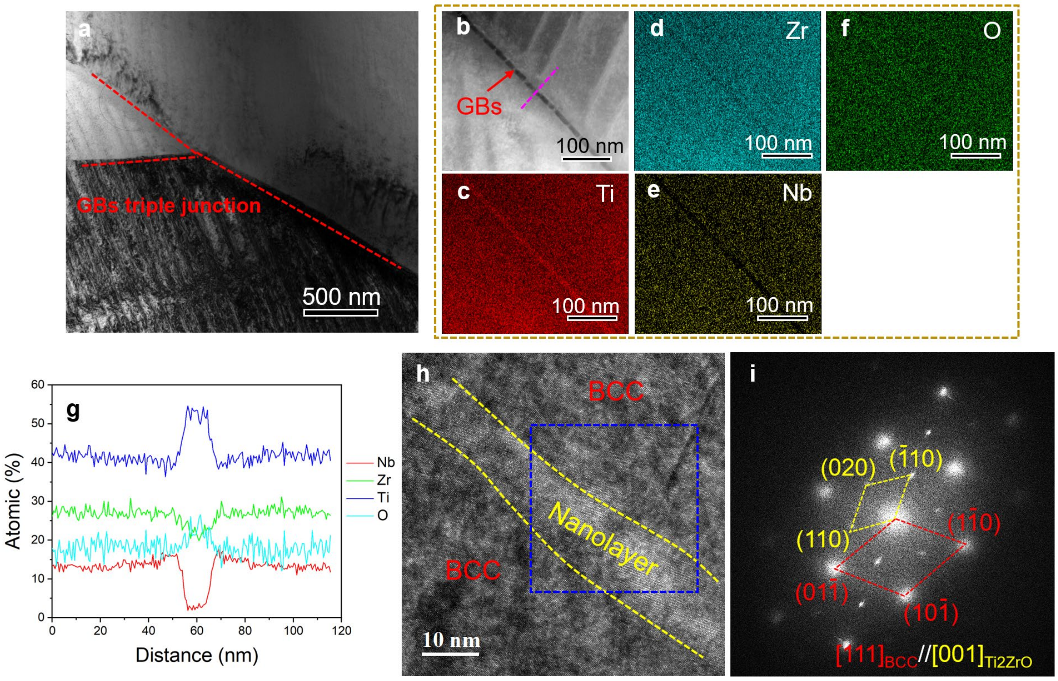

Supplementary Fig. 7 Grain boundary characterization of additively manufactured $Ti_{56}Zr_{30}Nb_{14}$ medium-entropy alloys. a, TEM image showing a grain boundary triple junction. **b**, Magnified view of the grain boundary region marked in (a). **c-f**, Corresponding energy dispersive spectroscopy (EDS) elemental mapping of the area marked in (b). **g**, Elemental line-scan profile acquired along the pink dashed line across the grain boundary in (b). **h**, High-resolution transmission electron microscopy (HRTEM) image of the grain boundary shown in (b). **i**, Selected area electron diffraction (SAED) pattern taken from the blue square region in (h).

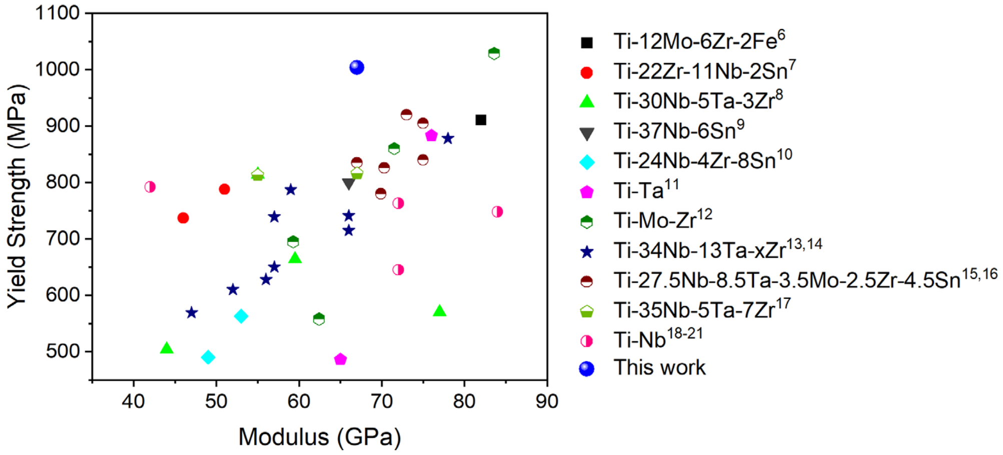

Supplementary Fig. 8 Strength-modulus of the AD3-AM MEA in comparison with state-of-the-art AM low-mudulus β Ti alloys[6-21].

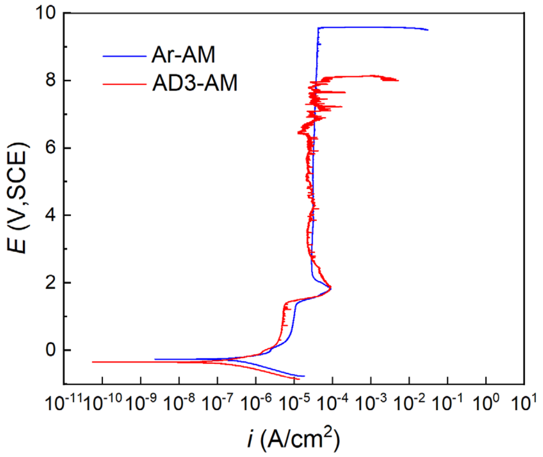

Supplementary Fig. 9 Corrosion resistance characterization of AM $Ti_{56}Zr_{30}Nb_{14}$ medium-entropy alloys via potentiodynamic polarization testing. Potentiodynamic polarization curves of the Ar-AM and optimally air-doped AD3-AM samples, tested in 0.9 wt.% NaCl electrolyte with a saturated calomel electrode (SCE) as the reference electrode.

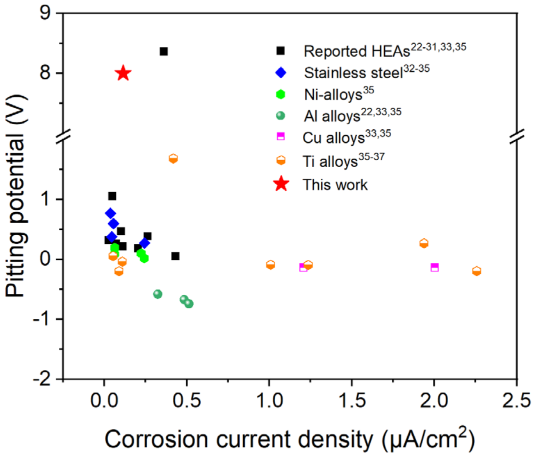

Supplementary Fig. 10 Relative comparison of corrosion resistance between the AD3-AM alloy and reported metallic alloys. Pitting potential versus corrosion current density for the optimally AD3-AM alloy, alongside previously reported high-entropy alloys and conventional passive alloys[22-37]. Literature data were obtained under different test conditions, and are provided for relative performance reference only. The plot confirms the outstanding corrosion resistance of the AD3-AM alloy, with a high pitting potential and low corrosion current density.

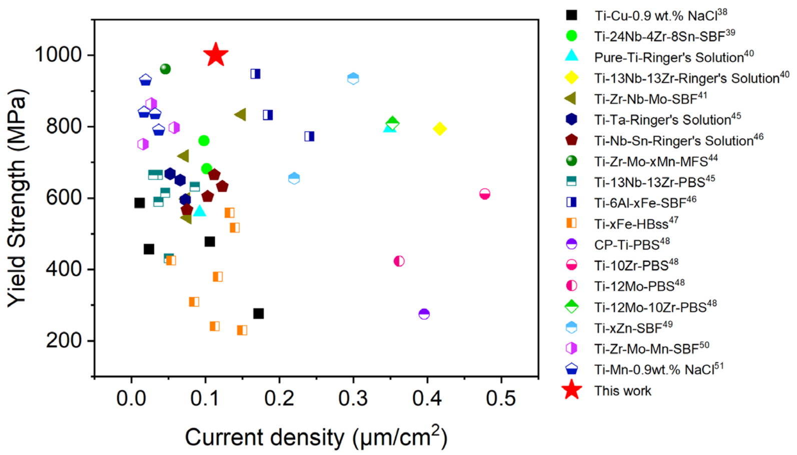

Supplementary Fig. 11 Comparison of the corrosion current density and yield strength of the AD3-AM medium-entropy alloy with those of most reported titanium alloys. Note that the reference data were obtained from different media and solution concentrations[38-51].

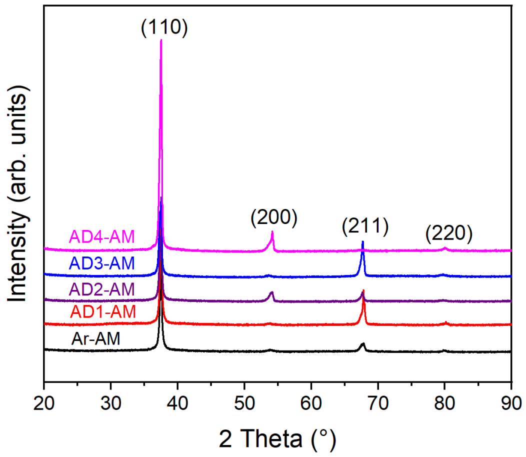

Supplementary Fig. 12 XRD patterns of AM samples processed under varying air-doping levels.

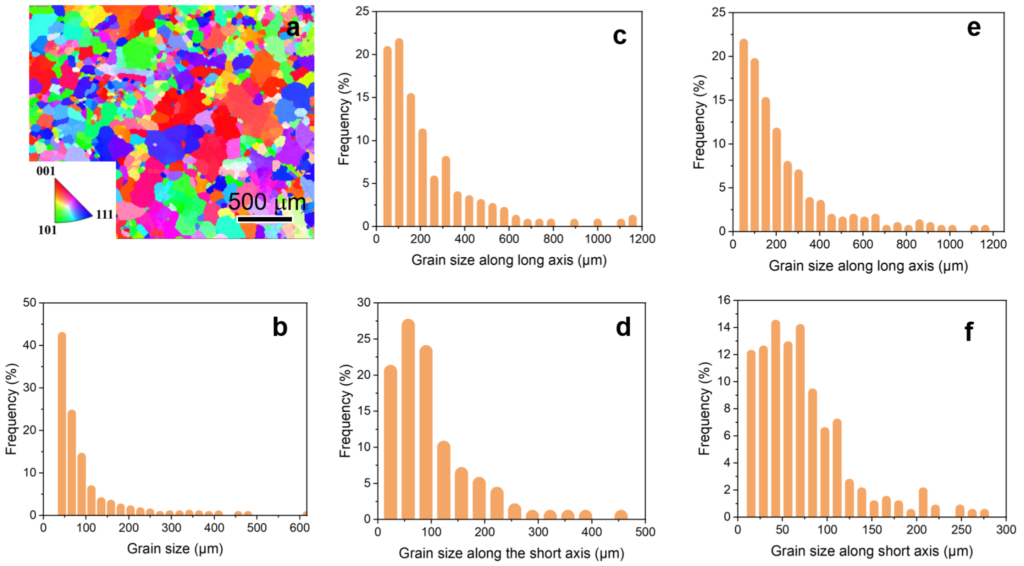

Supplementary Fig. 13 Grain structure and grain size distribution characterization of as-cast and AM $Ti_{56}Zr_{30}Nb_{14}$ medium-entropy alloys. a, Inverse Pole Figure (IPF) map acquired via electron backscatter diffraction (EBSD) of the as-cast alloy. **b**, Corresponding grain size distribution histogram of the as-cast sample. **c** and **d**, Grain size distribution histograms of the Ar-AM sample along the building direction (BD, longitudinal) and transverse direction (TD, perpendicular to BD), respectively. **e** and **f**, Grain size distribution histograms of the AD3-AM sample along the building direction and transverse direction, respectively.

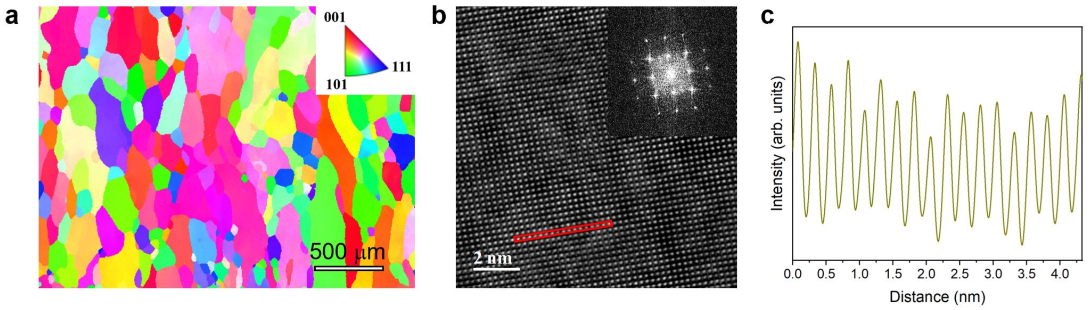

Supplementary Fig. 14 Microstructure characterization of the Ar-AM samples. a, EBSD IPF map along the deposition direction. **b**, HAADF-STEM image viewed along the [001] crystal axis; the inset shows the corresponding fast Fourier transform (FFT) pattern. **c**, Intensity line profile taken from the red squared region in (b).

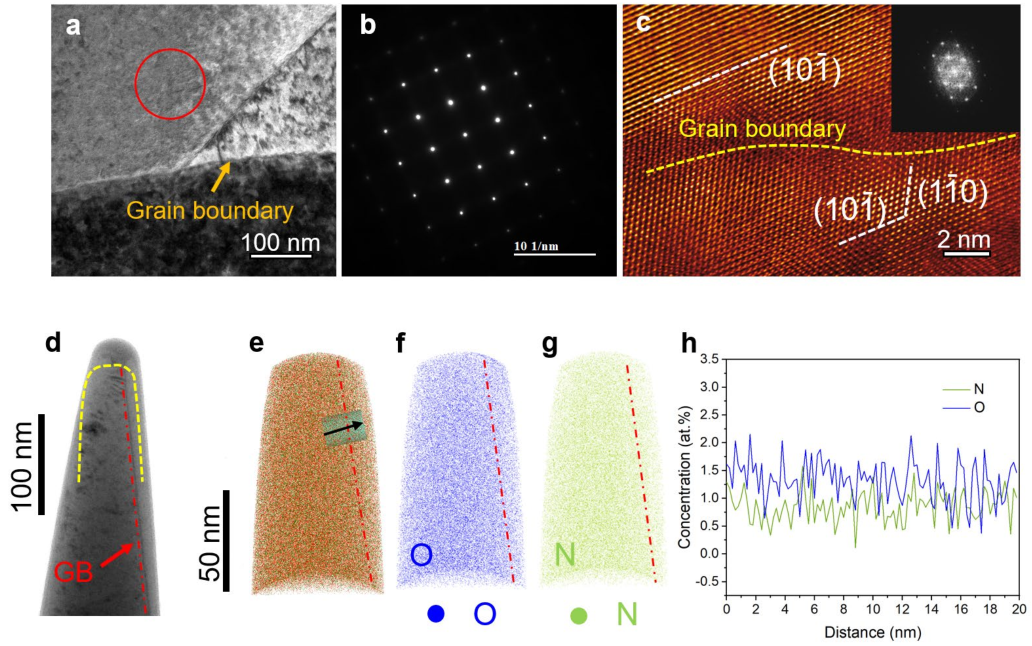

Supplementary Fig. 15 Grain boundary microstructure and interstitial element distribution of the AD3-AM alloy. a, TEM bright-field image, showing a clear grain boundary. **b**, SAED pattern acquired from the red circular region in (a). **c**, High-resolution transmission electron microscopy (HRTEM) atomic image of the grain boundary in (a); the inset shows the corresponding FFT pattern, confirming the clean grain boundary without second-phase precipitates. **d**, TEM bright-field image of theAPT tip specimen containing a grain boundary (marked as GB). **e**, Three-dimensional APT reconstruction of the analyzed volume from the tip specimen. **f** and **g**, Elemental distribution maps of O and N from the APT reconstruction, respectively. **h**, One-dimensional concentration profile of O and N elements across the grain boundary, extracted along the direction indicated by the black arrow in (e).

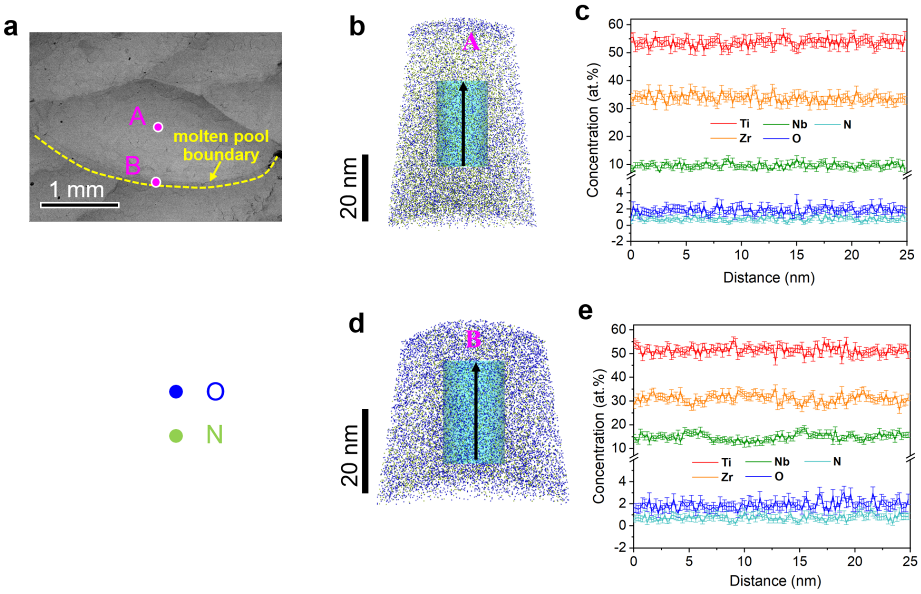

Supplementary Fig. 16 Homogeneity of interstitial element distribution at melt pool center and boundary in the AD3-AM alloy. a, SEM image showing the melt pool morphology of the AD3-AM sample, with the melt pool boundary outlined by the yellow dashed curve. **b**, APT reconstruction of O/N elements from region A (melt pool center marked in a), with O and N elemental distribution displayed. **c**, corresponding 1D concentration profile across the cylinder in b. **d**, APT reconstruction of O/N elements from region B (melt pool boundary marked in a), with O and N elemental distribution displayed. **e**, corresponding 1D concentration profile across the cylinder in d. Error bars correspond to the standard error (SE) of the APT concentration measurements.

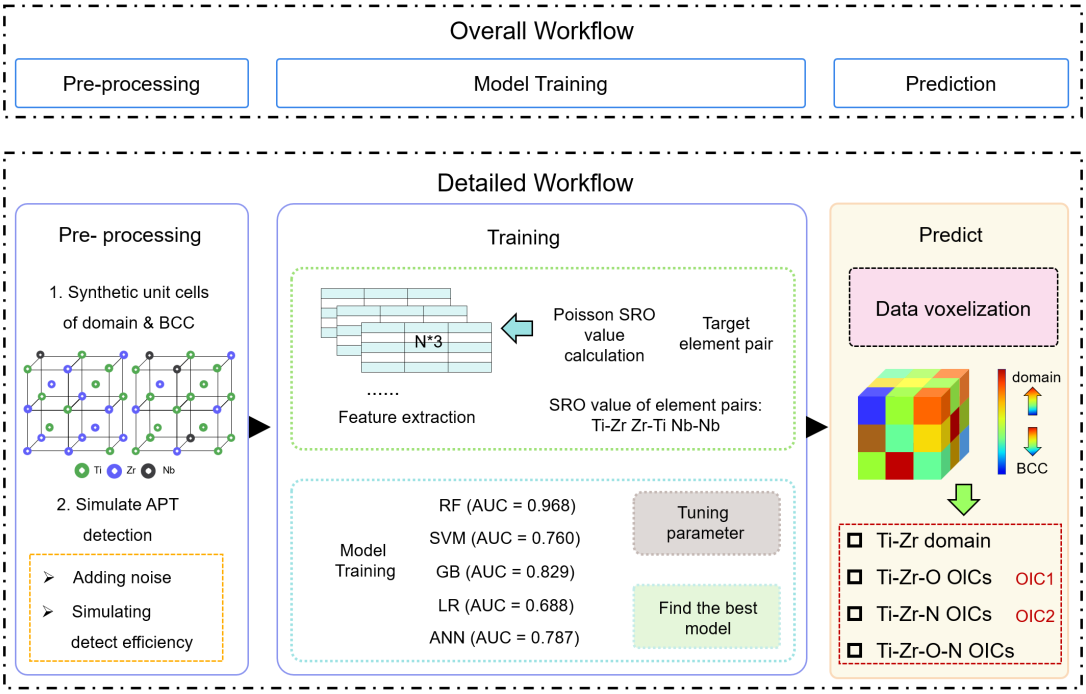

Supplementary Fig. 17 Schematic workflow of the proposed ML-APT framework for identification of multiple type OICs motifs in $Ti_{56}Zr_{30}Nb_{14}$ alloys.

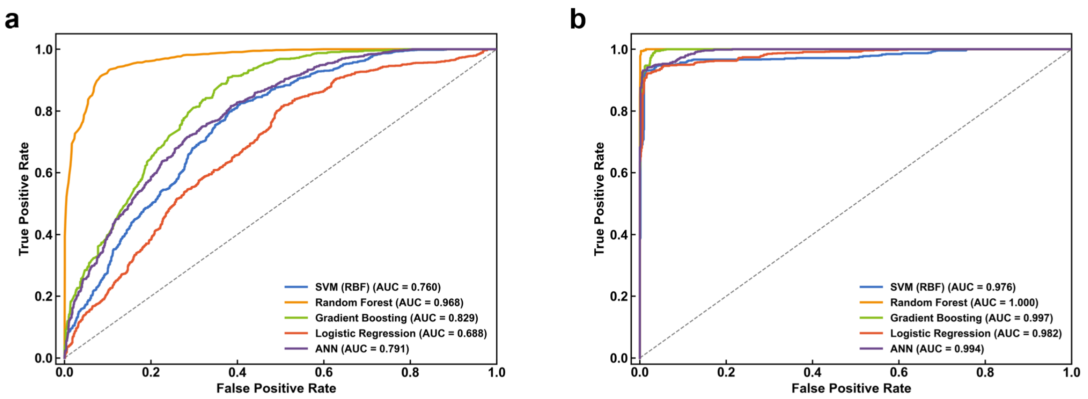

Supplementary Fig. 18 Comparison of ROC curves from different ML models. a, Ti-Zr-rich domains characterization model. **b**, Nb-rich domains characterization model.

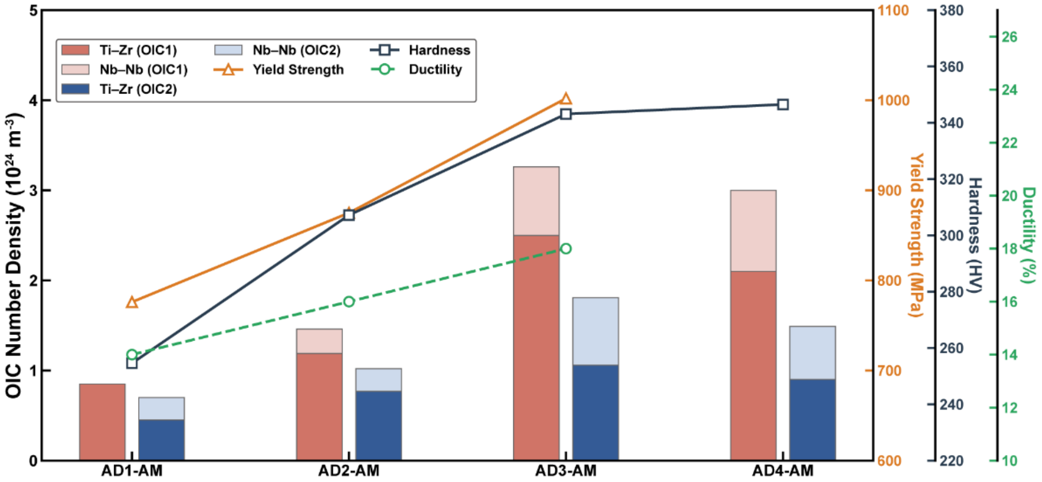

Supplementary Fig. 19 Number density of OICs and mechanical properties (hardness, yield strength, ductility) in different domains of the AM alloy under various atmosphere doping levels.

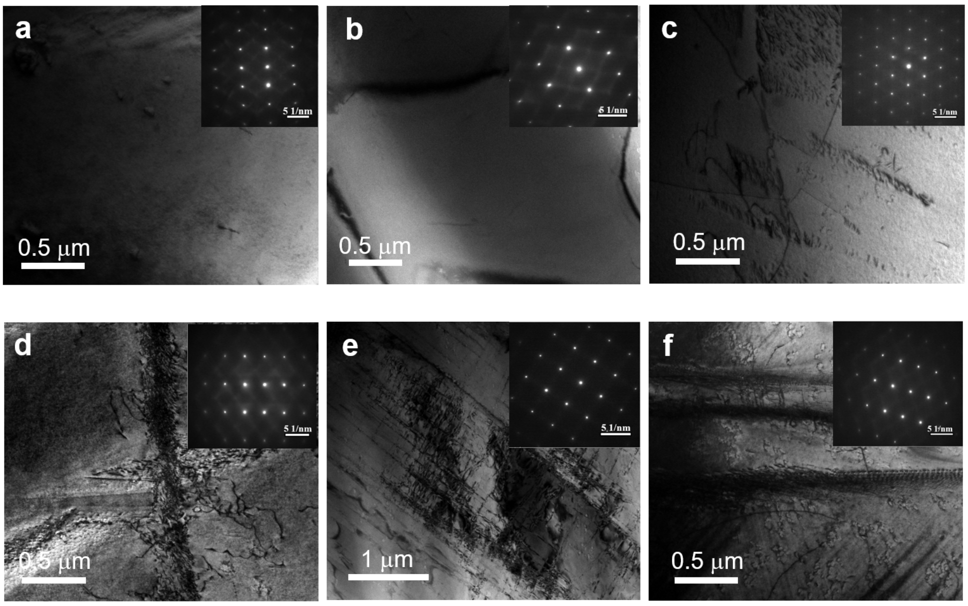

Supplementary Fig. 20 Microstructure evolution of AM alloys before and after tensile deformation. a-c, TEM images with SAED insets of the Ar-AM, AD2-AM and AD3-AM alloys before deformation, respectively. **d-f**, TEM images with SAED insets of the corresponding alloys after tensile fracture, respectively.

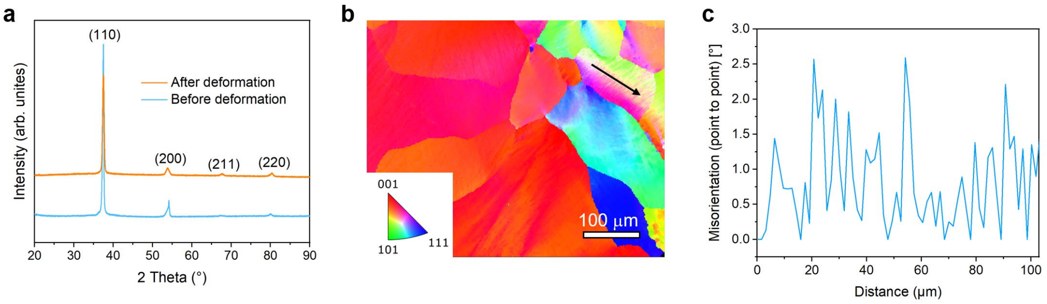

Supplementary Fig. 21 Phase and microstructure characterization of AD3-AM alloy before and after deformation. a, XRD patterns. **b**, EBSD IPF map. **c**, Point-to-point misorientation angle profile along the black arrow in (b).

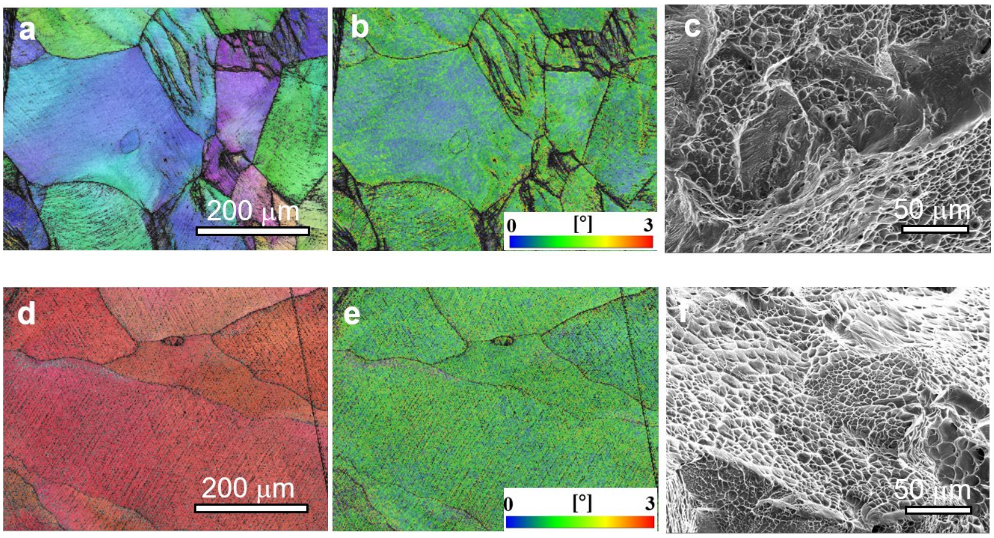

Supplementary Fig. 22 Deformed microstructure and tensile fracture morphology of Ar-AM and AD3-AM alloys. a and **b** EBSD IPF and KAM maps of the deformed Ar-AM sample. **c**, SEM tensile fracture surface of the Ar-AM sample. **d** and **e** EBSD IPF and KAM maps of the deformed AD3-AM sample. **f**, SEM tensile fracture surface of the AD3-AM sample.

Supplementary references

1. Neumann G, Tuijn C. Self-diffusion and impurity diffusion in pure metals: handbook of experimental data[M]. Elsevier, 2011.
2. Xia C, Shi Y, Li Y, et al. Study on microstructure and mechanical and corrosion properties of Zr-Ti-O ternary alloy[J]. Journal of Alloys and Compounds, 2025: 182053.
3. Li Y, Colnaghi T, Gong Y, et al. Machine learning‐enabled tomographic imaging of chemical short‐range atomic ordering[J]. Advanced materials, 2024, 36(44): 2407564.
4. Liu S, Covian A C, Wang X, et al. 3D nanoscale mapping of short‐range order in GeSn alloys[J]. Small Methods, 2022, 6(5): 2200029.
5. Li Y, Wei Y, Wang Z, et al. Quantitative three-dimensional imaging of chemical short-range order via machine learning enhanced atom probe tomography[J]. Nature Communications, 2023, 14(1): 7410.
6. Duan R, Li S, Cai B, et al. A high strength and low modulus metastable β Ti-12Mo-6Zr-2Fe alloy fabricated by laser powder bed fusion in-situ alloying[J]. Additive Manufacturing, 2021, 37: 101708.
7. Schaal H, Castany P, Laheurte P, et al. Design of a low Young's modulus Ti-Zr-Nb-Sn biocompatible alloy by in situ laser powder bed fusion additive manufacturing process[J]. Journal of Alloys and Compounds, 2023, 966: 171539.
8. Luo J P, Sun J F, Huang Y J, et al. Low-modulus biomedical Ti-30Nb-5Ta-3Zr additively manufactured by Selective Laser Melting and its biocompatibility[J]. Materials Science and Engineering: C, 2019, 97: 275-284.
9. Chen W, Chen C, Zi X, et al. Controlling the microstructure and mechanical properties of a metastable β titanium alloy by selective laser melting[J]. Materials Science and Engineering: A, 2018, 726: 240-250.
10. Zhang L C, Klemm D, Eckert J, et al. Manufacture by selective laser melting and mechanical behavior of a biomedical Ti-24Nb-4Zr-8Sn alloy[J]. Scripta Materialia, 2011, 65(1): 21-24.
11. Gao M, He D, Cui L, et al. Investigation on the Microstructure and Mechanical Properties of the Ti-Ta Alloy with Unmelted Ta Particles by Laser Powder Bed Fusion[J]. Materials, 2023, 16(6): 2208.
12. Liang R, Jiang Y, Tao J, et al. Customizing the Young's modulus of Ti-Mo-Zr alloys by in situ additive manufacturing based on Mo spatial concentration modulation[J]. Journal of Materials Research and Technology, 2024, 29: 5607-5619.
13. Kong W, Cox S C, Lu Y, et al. Microstructural evolution, mechanical properties, and preosteoblast cell response of a post-processing-treated TNT5Zr β Ti alloy manufactured via selective laser melting[J]. ACS Biomaterials Science & Engineering, 2022, 8(6): 2336-2348.
14. Kong W, Cox S C, Lu Y, et al. The influence of zirconium content on the microstructure, mechanical properties, and biocompatibility of in-situ alloying Ti-Nb-Ta based β alloys processed by selective laser melting[J]. Materials Science and Engineering: C, 2021, 131: 112486.
15. Alabort E, Tang Y T, Barba D, et al. Alloys-by-design: A low-modulus titanium alloy for additively manufactured biomedical implants[J]. Acta Materialia, 2022, 229: 117749.
16. Suwanpreecha C, Alabort E, Tang Y T, et al. A novel low-modulus titanium alloy for biomedical applications: a comparison between selective laser melting and metal injection moulding[J]. Materials Science and Engineering: A, 2021, 812: 141081.
17. Gao Y, Jiang W, Zeng D, et al. Additive manufacturing of titanium alloys for biomedical applications: A systematic review[J]. Review of Materials Research, 2025: 100011.
18. Fischer M, Joguet D, Robin G, et al. In situ elaboration of a binary Ti-26Nb alloy by selective laser melting of elemental titanium and niobium mixed powders[J]. Materials Science and Engineering: C, 2016, 62: 852-859.
19. Zhang J W, Dong Y P, Tang J C, et al. Additive manufacturing of the high-strength and low modulus biomedical Ti-10 Nb alloy under reactive atmosphere[J]. Materials Today Communications, 2022, 33: 104837.

20. Wang J, Liu Y, Rabadia C D, et al. Microstructural homogeneity and mechanical behavior of a selective laser melted Ti-35Nb alloy produced from an elemental powder mixture[J]. Journal of Materials Science & Technology, 2021, 61: 221-233.
21. Pilz S, Gustmann T, Günther F, et al. Controlling the Young's modulus of a β-type Ti-Nb alloy via strong texturing by LPBF[J]. Materials & Design, 2022, 216: 110516.
22. Qiu Y, Thomas S, Gibson M A, et al. Corrosion of high entropy alloys[J]. npj Materials degradation, 2017, 1(1): 15. HEA.
23. Shi Y, Yang B, Liaw P K. Corrosion-resistant high-entropy alloys: a review. Metals 7: 43[EB/OL].(2017).
24. Shi Y, Yang B, Xie X, et al. Corrosion of AlxCoCrFeNi high-entropy alloys: Al-content and potential scan-rate dependent pitting behavior[J]. Corrosion Science, 2017, 119: 33-45. HEA
25. Lin C M, Tsai H L. Evolution of microstructure, hardness, and corrosion properties of high-entropy Al0.5CoCrFeNi alloy[J]. Intermetallics, 2011, 19(3): 288-294. HEA
26. Hsu Y J, Chiang W C, Wu J K. Corrosion behavior of FeCoNiCrCux high-entropy alloys in 3.5% sodium chloride solution[J]. Materials Chemistry and Physics, 2005, 92(1): 112-117. HEA
27. Wu C L, Zhang S, Zhang C H, et al. Phase evolution and cavitation erosion-corrosion behavior of FeCoCrAlNiTix high entropy alloy coatings on 304 stainless steel by laser surface alloying[J]. Journal of Alloys and Compounds, 2017, 698: 761-770. Stainless steel
28. Zhang S, Wu C L, Zhang C H, et al. Laser surface alloying of FeCoCrAlNi high-entropy alloy on 304 stainless steel to enhance corrosion and cavitation erosion resistance[J]. Optics & Laser Technology, 2016, 84: 23-31. HEA
29. Shang C, Axinte E, Sun J, et al. CoCrFeNi(W1-xMox) high-entropy alloy coatings with excellent mechanical properties and corrosion resistance prepared by mechanical alloying and hot pressing sintering[J]. Materials & Design, 2017, 117: 193-202. HEA
30. Ye Q, Feng K, Li Z, et al. Microstructure and corrosion properties of CrMnFeCoNi high entropy alloy coating[J]. Applied Surface Science, 2017, 396: 1420-1426. HEA
31. Qiu X. Microstructure, hardness and corrosion resistance of Al2CoCrCuFeNiTix high-entropy alloy coatings prepared by rapid solidification[J]. Journal of Alloys and Compounds, 2018, 735: 359-364. HEA
32. shankar Dhandapani V, Thangavel E, Arumugam M, et al. Effect of Ag content on the microstructure, tribological and corrosion properties of amorphous carbon coatings on 316L SS[J]. Surface and Coatings Technology, 2014, 240: 128-136. Stainless steel
33. Qiu Y, Gibson M A, Fraser H L, et al. Corrosion characteristics of high entropy alloys[J]. Materials science and technology, 2015, 31(10): 1235-1243. HEA
34. Pardo A, Merino M C, Coy A E, et al. Pitting corrosion behaviour of austenitic stainless steels–combining effects of Mn and Mo additions[J]. Corrosion Science, 2008, 50(6): 1796-1806. Stainless steel
35. Zhou Q, Sheikh S, Ou P, et al. Corrosion behavior of Hf0.5Nb0.5Ta0.5Ti1.5Zr refractory high-entropy in aqueous chloride solutions[J]. Electrochemistry Communications, 2019, 98: 63-68.
36. Lin J, Ozan S, Munir K, et al. Effects of solution treatment and aging on the microstructure, mechanical properties, and corrosion resistance of a β type Ti-Ta-Hf-Zr alloy[J]. RSC advances, 2017, 7(20): 12309-12317.
37. Vasilescu C, Drob S I, Moreno J M C, et al. Long-term corrosion resistance of new Ti-Ta-Zr alloy in simulated physiological fluids by electrochemical and surface analysis methods[J]. Corrosion Science, 2015, 93: 310-323.
38. Wang J, Zhang S, Sun Z, et al. Optimization of mechanical property, antibacterial property and corrosion resistance of Ti-Cu alloy for dental implant[J]. Journal of materials science & technology, 2019, 35(10): 2336-2344.

39. Li X, Tang J, Ju J, et al. Superior corrosion resistance and good biocompatibility of Ti-24Nb-4Zr-8Sn alloy fabricated by a cost-effective, net-shape powder metallurgy method[J]. Materials Futures, 2025, 4(3): 035401.
40. Zhou L, Yuan T, Tang J, et al. Mechanical and corrosion behavior of titanium alloys additively manufactured by selective laser melting-A comparison between nearly β titanium, α titanium and α+ β titanium[J]. Optics & Laser Technology, 2019, 119: 105625.
41. Chui P, Jing R, Zhang F, et al. Mechanical properties and corrosion behavior of β-type Ti-Zr-Nb-Mo alloys for biomedical application[J]. Journal of Alloys and Compounds, 2020, 842: 155693.
42. Zhao D, Han C, Li Y, et al. Improvement on mechanical properties and corrosion resistance of titanium-tantalum alloys in-situ fabricated via selective laser melting[J]. Journal of Alloys and Compounds, 2019, 804: 288-298.
43. Moraes P E L, Contieri R J, Lopes E S N, et al. Effects of Sn addition on the microstructure, mechanical properties and corrosion behavior of Ti-Nb-Sn alloys[J]. Materials Characterization, 2014, 96: 273-281
44. Liu H, Yang J, Zhao X, et al. Microstructure, mechanical properties and corrosion behaviors of biomedical Ti-Zr-Mo-xMn alloys for dental application[J]. Corrosion Science, 2019, 161: 108195.
45. Hariharan A, Goldberg P, Gustmann T, et al. Designing the microstructural constituents of an additively manufactured near β Ti alloy for an enhanced mechanical and corrosion response[J]. Materials & Design, 2022, 217: 110618.
46. Lu J, Zhao Y, Niu H, et al. Electrochemical corrosion behavior and elasticity properties of Ti-6Al-xFe alloys for biomedical applications[J]. Materials Science and Engineering: C, 2016, 62: 36-44.
47. Niu J, Guo Y, Li K, et al. Improved mechanical, bio-corrosion properties and in vitro cell responses of Ti-Fe alloys as candidate dental implants[J]. Materials Science and Engineering: C, 2021, 122: 111917.
48. Guo Z, Huang Y, Sun C, et al. Ti-Mo-Zr alloys for bone repair: mechanical properties, corrosion resistance, and biological performance[J]. journal of materials research and technology, 2023, 24: 7624-7637.
49. Chen Y T, Xu J L, Huang J, et al. Microstructure, mechanical properties, and corrosion resistance of biomedical Ti-Zn alloys prepared by spark plasma sintering[J]. Intermetallics, 2025, 185: 108926.
50. Li Z, Wo J, Fu Y, et al. Effects of Zr addition on the microstructural evolution, mechanical properties, and corrosion behavior of novel biomedical Ti-Zr-Mo-Mn alloys[J]. ACS Biomaterials Science & Engineering, 2023, 9(12): 6935-6946.
51. Zhou X, Fang H, Yuan T, et al. Effect of slight Sn modification on mechanical properties and corrosion behavior of Ti-(2-4 wt%) Mn alloys fabricated via powder metallurgy[J]. Materials Characterization, 2023, 203: 113068.
