# Supplementary material for: Strength-ductility synergy in medium-entropy alloys via harnessing trace air in additive manufacturing
Source: Nat Commun. 2026 Apr 29;17:5870. doi: 10.1038/s41467-026-72511-8 (PMC13333971; doi:10.1038/s41467-026-72511-8)
Supplement: Supplementary file 3 — Transparent Peer Review file [file 41467_2026_72511_MOESM3_ESM.pdf]

nature portfolio

Peer Review File

Strength-Ductility Synergy in Medium-Entropy Alloys via Harnessing Trace Air in Additive Manufacturing

Corresponding Author: Professor Zhaoping Lu

This file contains all reviewer reports in order by version, followed by all author rebuttals in order by version.

Version 0:

Reviewer comments:

Reviewer #1

(Remarks to the Author)
The authors present a compelling study on using trace oxygen and nitrogen from air during the additive manufacturing of a Ti–Zr–Nb medium-entropy alloy to achieve an exceptional strength–ductility synergy. By controllably introducing air into the build atmosphere, they facilitate the in-situ formation of nanoscale ordered interstitial complexes that enhance both strength and ductility, which can effectively overcome the conventional trade-off. The manuscript is strengthened by the sophisticated use of characterization techniques, especially machine-learning-assisted atom probe tomography, to identify and classify the OICs. Well-structured and supported by robust data, the work has significant implications for high-performance metal additive manufacturing. I will strongly recommend its publication after minor revisions to address the following points:
1. The manuscript establishes that the incorporation of oxygen and nitrogen is central to the formation of OICs and the resulting mechanical performance. To strengthen this foundation, please provide a quantitative discussion of the oxygen content in each sample. Including specific measurements would help readers better correlate the degree of air doping with the microstructural and mechanical properties observed.
2. The number density of OIC1 and OIC2 increases with air doping, yet the mechanical properties peak at AD3 and decline at AD4. The authors attribute the degradation at AD4 to grain boundary segregation, but no direct evidence is provided. Please include supporting data to substantiate this claim.
3. The manuscript identifies four distinct types of Ti-Zr-rich and Nb-rich domains. A more detailed discussion on the specific roles of each domain type plays in the reinforcement mechanism would significantly strengthen the analysis.

(Remarks on code availability)

Reviewer #3

(Remarks to the Author)
This study offers significant insights into addressing the challenges associated with conventional metal additive manufacturing, such as the high operational and maintenance costs and the narrow processing window imposed by the requirement for high-vacuum or ultra-high-purity inert gas atmospheres. For the first time, the authors proactively introduced readily available and inexpensive trace air into the build chamber during the AM process. By precisely controlling the air content, they achieved tailored microstructural characteristics and mechanical properties, resulting in an exceptional synergy between strength and ductility. A newly developed ML algorithm coupled with APT uncovers two dominant OICs structures and clarifies their role in deformation. The work is highly innovative and opens a fresh avenue for both materials science and AM. I recommend publication in Nature Communications after major revision.

1. Figure 1(a) clearly shows that, compared to both the as-cast condition and the alloy printed under high-purity argon (Ar-AM), the alloy printed with the optimal air doping content (AD3-AM) achieves an exceptional synergistic improvement in both strength and ductility. Here, a question arises: why does the performance of Ar-AM exceed that of the as-cast alloy? and why is AD4-AM so brittle? Please explain.

2. When emphasizing the anomalous strength-ductility synergy, the authors compared three alloys: the as-cast alloy, the Ar-AM alloy, and the AD3-AM alloy. Although IPF maps illustrating the grain morphology of the Ar-AM and AD3-AM alloys are

provided in Fig. 2b and Supplementary Fig. 8a, respectively, the IPF map of the as-cast alloy is missing and should be supplemented. Additionally, the grain size distribution should also be provided to allow for a more intuitive understanding of the macroscopic microstructural characteristics.

3. Recently, some studies have reported on oxygen addition in additively manufactured titanium alloys [Song T, Chen Z, Cui X, et al. Strong and ductile titanium-oxygen-iron alloys by additive manufacturing. Nature, 2023, 618(7963): 63-68]. While the effectiveness of actively utilizing air for performance tuning in this work is evident, how does this atmosphere-mediated impurity functionalization approach compare with the route of directly adding oxide powders (such as TiO_2, ZrO_2, etc.) during printing in terms of mechanical properties? Which one is superior? It is recommended that the authors delve into the advantages of the new pathway from a processing perspective, providing an in-depth discussion with clear logic and reasoning.

4. The absence of cracks in the alloy after printing with air doping demonstrates its excellent printability. However, porosity also significantly impacts mechanical properties. Although the authors mention that the bulk relative density measured by CT reaches 99.99%, the size and distribution of pores were not provided. It is therefore recommended that the authors supply this important information.

5. The repeated thermal cycling effect indeed decomposes the initially surface contamination films into oxygen and nitrogen atoms in solid solution, which represents a distinct advantage of the additive manufacturing process. However, are these elements completely dissolved within the grains, or is there some segregation at the grain boundaries? Oxygen and nitrogen atoms are uniformly distributed at the surface and inside the molten pool? It is well-known that impurity segregation at grain boundaries and the surface of the molten pool often has detrimental effects on mechanical properties. The authors are encouraged to provide high-resolution TEM images or atom probe tomography (APT) data of the grain boundaries to verify this.

6. The authors state in the main text: “The AD3-AM MEA meets all three requirements: its breakdown potential reaches 8 V, far surpassing conventional Ti alloys”. It is indeed remarkable to demonstrate such corrosion resistance alongside a yield strength exceeding 1 GPa. However, could the authors supplement this claim with a performance comparison plot against conventional titanium alloys to make the argument more compelling?

7. The repeated thermal cycling process of AM also results in differences in microstructures and mechanical properties at different heights. How in this work? In addition, anisotropic microstructure and mechanical properties are common during the AM. they can be considered.

8. How to quantify the content of oxygen and nitrogen dissolved in the alloy?

(Remarks on code availability)

Version 1:

Reviewer comments:

Reviewer #1

(Remarks to the Author)

(Remarks on code availability)

Reviewer #3

(Remarks to the Author)
This work holds significance for the additive manufacturing of metallic parts and broadens the processing window. All comments have been addressed, and the manuscript quality meets the standards for publication.

(Remarks on code availability)

Open Access This Peer Review File is licensed under a Creative Commons Attribution 4.0 International License, which permits use, sharing, adaptation, distribution and reproduction in any medium or format, as long as you give appropriate credit to the original author(s) and the source, provide a link to the Creative Commons license, and indicate if changes were made.
In cases where reviewers are anonymous, credit should be given to 'Anonymous Referee' and the source.
The images or other third party material in this Peer Review File are included in the article’s Creative Commons license, unless indicated otherwise in a credit line to the material. If material is not included in the article’s Creative Commons license and your intended use is not permitted by statutory regulation or exceeds the permitted use, you will need to obtain permission directly from the copyright holder.
To view a copy of this license, visit https://creativecommons.org/licenses/by/4.0/

Point-by-point responses

We sincerely thank the reviewers for providing invaluable guidance and positive feedback on our manuscript titled "**Anomalous Strength-Ductility Synergy in Medium-Entropy Alloys via Harnessing Trace Air in Additive Manufacturing**". Below, we have provided point-by-point responses to each specific comment from the reviewers. The reviewer comments are in **red**, and replies in **black** and modifications to the manuscript quoted here in **blue**.

Reviewer: 1

The authors present a compelling study on using trace oxygen and nitrogen from air during the additive manufacturing of a Ti-Zr-Nb medium-entropy alloy to achieve an exceptional strength-ductility synergy. By controllably introducing air into the build atmosphere, they facilitate the in-situ formation of nanoscale ordered interstitial complexes that enhance both strength and ductility, which can effectively overcome the conventional trade-off. The manuscript is strengthened by the sophisticated use of characterization techniques, especially machine-learning-assisted atom probe tomography, to identify and classify the OICs. Well-structured and supported by robust data, the work has significant implications for high-performance metal additive manufacturing. I will strongly recommend its publication after minor revisions to address the following points.

Reply: We are truly delighted by the reviewer's favorable evaluation and are greatly encouraged by their supportive feedback.

1. The manuscript establishes that the incorporation of oxygen and nitrogen is central to the formation of OICs and the resulting mechanical performance. To strengthen this foundation, please provide a quantitative discussion of the oxygen content in each sample. Including specific measurements would help readers better correlate the degree of air doping with the microstructural and mechanical properties observed.

Reply: We thank the reviewer for this constructive suggestion. We agree that a clear quantitative correlation between the air-doping level and the resulting properties is essential. As compelling evidence, in addition to Supplementary Fig. 3, we present in **Table R1** and **Fig. R1** the key quantitative data, which list the oxygen/nitrogen content, number density of OICs, hardness, yield strength and ductility for all samples from AD1-AM to AD4-AM. Specifically, the oxygen content increases from 0.20 wt.% (AD1-AM), 0.25 wt.% (AD2-AM), 0.33 wt.% (AD3-AM) to 0.40 wt.% (AD4-AM), while nitrogen rises correspondingly from 0.068 wt.% to 0.28 wt.%. From AD1- to AD3-AM, the data reveal a clear and systematic trend: with increasing air-doping level (and thus higher oxygen/nitrogen content), both the number density of OICs and the mechanical properties increase. For instance, the OIC1 density in Ti-Zr domains rises from 0.85×10^{24} m^{-3} to 2.50×10^{24} m^{-3}, yield strength increases from 776 MPa to 1002 MPa, hardness from 254.6 HV to 343.1 HV, and ductility from 14% to 18%. Notably, the AD3-AM sample, containing 0.33 wt.% O and 0.18 wt.% N, achieves a yield

strength exceeding 1 GPa while maintaining ~18% tensile ductility. This sample represents an optimal doping window where interstitial solutes predominantly contribute to intragranular OIC formation. This provides direct quantitative evidence that the enhanced mechanical performance is intrinsically correlated with the controlled introduction of interstitial solutes.

This compositional trend is directly linked to the evolution of OICs, as supported by complementary microstructural analysis (e.g., **Fig. R1**). The increasing interstitial solute concentration from AD1 to AD3 promotes the formation and increased number density of beneficial OICs within the grains, which is the fundamental microstructural origin of the observed strength-ductility synergy. Furthermore, the performance degradation at AD4-AM, despite its highest interstitial content (0.4 wt.% O, 0.28 wt.% N), can be rationalized by a shift in the solute distribution. At this excessive doping level, a significant fraction of interstitials segregates at grain boundaries (as directly evidenced by APT in **Fig. R2**), leading to embrittlement rather than contributing to beneficial intragranular OIC formation. This accounts for both the hardness plateau at 346.5 HV and the fact that AD4-AM fractures before yielding in tension. In summary, the quantitative data in **Table R1** and **Fig. R1** establish a direct correlation between the air-doping level and the macroscopic mechanical properties, and this correlation is underpinned by a transition from beneficial intragranular OIC formation to detrimental grain-boundary segregation. This analysis has been integrated into the revised manuscript to strengthen the composition-structure-property discussion.

Table R1. Absorbed O/N content, domain-specific OIC number density, and mechanical properties of the AM alloy under various atmosphere doping levels.

Samples	Oxygen (wt.%)	Nitrogen (wt.%)	Number density (10^{24} m^{-3})				Hardness (HV)	Yield strength (MPa)	Ductility (%)
			Ti-Zr domains		Nb-Nb domains				
			OIC1	OIC2	OIC1	OIC2			
AD1-AM	0.20	0.068	0.85	0.45	-	0.25	254.6	776	14%
AD2-AM	0.25	0.14	1.19	0.77	0.27	0.25	307.2	875	16%
AD3-AM	0.33	0.18	2.50	1.06	0.76	0.75	343.1	1002	18%
AD4-AM	0.40	0.28	2.10	0.90	0.90	0.59	346.5	-	-

To comply with the reviewer’s comments, we have made the following specific revisions to the manuscript:

Manuscript (Page 13 line 9 to Page 14 line 8)

Furthermore, in the Ti-Zr-rich domains, the number densities of OIC1 (O-Zr-Ti) and OIC2 (N-Zr-Ti) evolve systematically with increasing air-doping level. At lower doping stages (AD1 and AD2), their densities remain relatively low. When the doping level increases from AD2 to AD3, the OIC1 number density more than doubles—from 1.19×10^{24} m^{-3} to 2.50×10^{24} m^{-3}—while OIC2 also shows a concurrent rise from 0.77 to 1.06×10^{24} m^{-3}. However, in the excessively doped AD4 sample, both OIC1 and

OIC2 densities decrease to 2.10 and 0.90 × 10^{24} m^{-3}, respectively. In Nb-rich domains, OICs remain sparse at lower doping levels; at the AD3 stage, both OIC1 (O-Nb) and OIC2 (N-Nb) densities rise to 0.76 and 0.75 × 10^{24} m^{-3}, and at AD4 the OIC1 density further increases to 0.90 × 10^{24} m^{-3} while OIC2 shows a decrease to 0.59 × 10^{24} m^{-3}, yet they remain substantially lower than those in the Ti-Zr-rich domains (Supplementary Fig. 19).

Correspondingly, the mechanical properties improve systematically up to the AD3 doping level. As shown in Supplementary Fig. 2, with the increase in total O/N content from 0.268 wt.% (AD1-AM) to 0.510 wt.% (AD3-AM), the yield strength rises from 776 MPa to 1002 MPa, the elongation improves from 14% to 18%, and the hardness increases from 254.6 HV to 343.1 HV. By intensifying lattice distortion (for solid-solution strengthening) and promoting the formation of intragranular OICs, elevated interstitial content thus underpins the observed strength–ductility synergy. However, at the highest doping level (AD4-AM), mechanical performance deteriorates due to pronounced segregation of excess interstitials at grain boundaries (Supplementary Fig. 5). This leads to embrittlement rather than further beneficial OIC formation, accounting for the plateau in hardness (346.5 HV) and the loss of ductility. The straightforward control of microstructure and resulting properties, achieved simply by adjusting the air-doping level in the build chamber, demonstrates exceptional tunability.

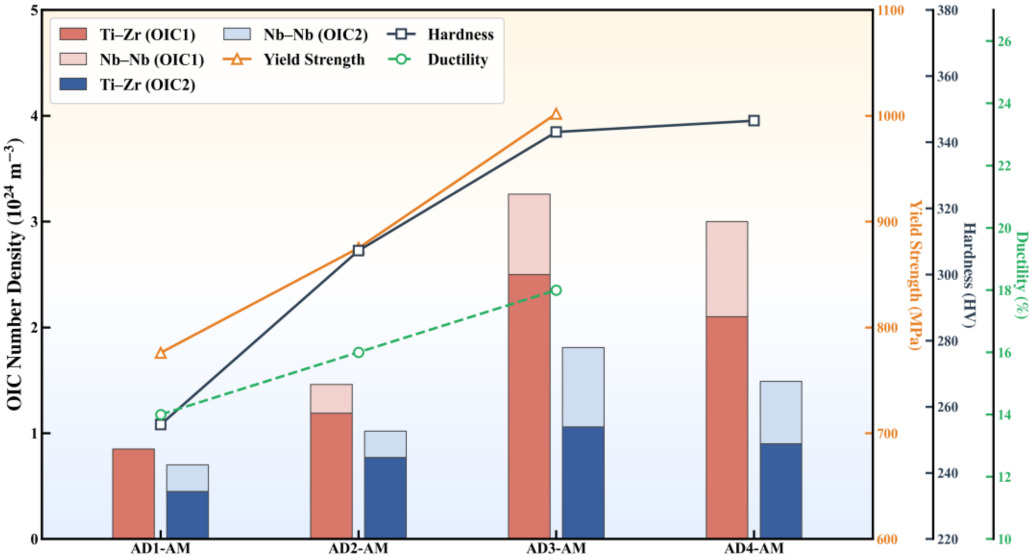

Fig. R1 Number density of OICs and mechanical properties (hardness, yield strength, ductility) in different domains of the AM alloy under various atmosphere doping levels.

2. The number density of OIC1 and OIC2 increases with air doping, yet the mechanical properties peak at AD3 and decline at AD4. The authors attribute the degradation at AD4 to grain boundary segregation, but no direct evidence is provided. Please include supporting data to substantiate this claim.

Reply: We thank the reviewer for highlighting the need for direct evidence regarding grain-boundary segregation in the AD4-AM sample. To provide direct evidence, we

performed APT on a tip specimen containing a grain boundary. As displayed in **Fig. R2a**, the oxygen isosurface clearly reveals enrichment of oxygen at the grain boundary. The corresponding 1D concentration profile (**Fig. R2b**) further confirms noticeable O and N segregation along the grain boundary. These results substantiate that excessive interstitial accumulation at grain boundaries in the AD4-AM condition leads to the observed loss of ductility.

Following the reviewer's suggestions, the manuscript has been revised as detailed below.

Manuscript (Page 5 lines 14-15)

as directly evidenced by APT (see Supplementary Fig. 5).

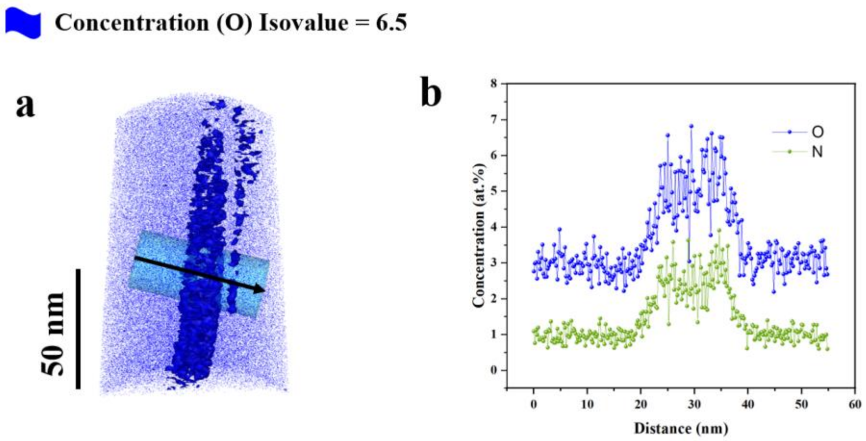

Fig. R2 a, Oxygen isoconcentration surface of 6.5% in the APT specimen containing a grain boundary. **b**, 1D concentration profile along the position marked by the black arrow in (a).

3. The manuscript identifies four distinct types of Ti-Zr-rich and Nb-rich domains. A more detailed discussion on the specific roles of each domain type plays in the reinforcement mechanism would significantly strengthen the analysis.

Reply: We thank the reviewer for raising this important point regarding the specific roles of the identified domain types. Following the reviewer's advice, we have added detailed discussion on the specific roles of each domain types. The four types function synergistically: Type 1 (pure metal-enriched domains) constitutes the inert matrix; Types 2 (OIC1) and 3 (OIC2) act as strong, correlated obstacles that provide additional strengthening and, importantly, promote dislocation cross-slip to enhance ductility; Type 4 (trace complexes) has a negligible influence. These points have been incorporated into the revised manuscript as summarized below.

Manuscript (Page 11 line 8 to Page 13 line 8)

Based on ML-APT analysis and mechanical modeling, a detailed quantitative discussion follows to clarify the individual contributions to the reinforcement. In the AD3-AM alloy, the increase in yield strength stems from the O/N interstitials (2.24 at.%), whose lattice distortion strongly interacts with dislocations [18,62].

Consequently, the classical interstitial solid-solution strengthening model is employed to quantify the contribution of O/N atoms, as shown in Eq. 1 [19]:

$$\Delta\tau = \frac{G\Delta\epsilon c^{1/2}}{3} \qquad (1)$$

Where G is the shear modulus (taken as 23.7 GPa according to [19]), $\Delta\epsilon$ represents the lattice mismatch parameter corresponding to the distortion induced by interstitial atoms (with values of 0.1 for O and 0.14 for N, respectively [17]), and c denotes the atomic concentration of interstitial atoms in the solid solution. Classical solid-solution modeling attributes ~257 MPa to these atoms, of which randomly dissolved interstitials (~90% of the total) contribute ~231 MPa.

While approximately 90% of the randomly distributed interstitial atoms constitute the fundamental basis for alloy strengthening, the remaining ~10% incorporated into OICs serve as the critical catalyst for achieving a significant leap in mechanical performance. These OICs exhibit distinctly different contribution logics dictated by the chemical characteristics of their central atomic domains. By considering the two primary chemical environments (Ti-Zr-rich and Nb-rich domains), four sub-types of OICs are identified and quantified. Type 2 (OIC1) accounts for approximately 6.5% of the total interstitials: the Ti-Zr domains OIC1, possessing the highest occupancy (~4.2%), contributes larger to strengthening comparing with the smaller Nb-Nb domains OIC1 (~2.3%). Crucially, these specific structures effectively promote dislocation cross-slip and enhance dislocation storage capacity [18], and thus contribute to the high ductility [21]. In contrast, type 3 (OIC2) encompasses approximately 3.5% of the interstitials, where the Ti-Zr domains (~2.1%) and the Nb-Nb domains (~1.4%) fractions contribute a moderate strength increment and supplementary reinforcement, respectively. This synergistic combination further increases the alloy's strength without sacrificing its ductility [21,63].

Therefore, the reinforcement mechanism emerges from a synergistic interplay of four domain types: the randomly dissolved interstitials (~90%) provide the solid solution strengthening; the ordered OICs (Types 2 & 3, ~10%) act as potent, correlated obstacles that deliver additional strengthening and ductility enhancement; the pure metal domains (Type 1) form the inert matrix; and the negligible Type 4 domains have minimal influence. In-depth analysis from physical modeling reveals that classical point-obstacle models predict a total theoretical strength lower than experimental values, this discrepancy precisely illuminates the strengthening essence of OICs as finite-sized obstacles. Distinct from the isolated solute point obstacles in traditional models, OICs—with a radius of approximately 0.7 nm—possess significant internal order, which induces a more potent synergistic stress field within the lattice [64,65].

Reference in the manuscript

[62] Yu Q, Qi L, Tsuru T, et al. Origin of dramatic oxygen solute strengthening effect in titanium[J]. Science, 2015, 347(6222): 635-639.

[63] Lei Z, Wu Y, He J, et al. Snoek-type damping performance in strong and ductile high-entropy alloys[J]. Science advances, 2020, 6(25): eaba7802.
[64] Chen X, Wang Q, Cheng Z, et al. Direct observation of chemical short-range order in a medium-entropy alloy[J]. Nature, 2021, 592(7856): 712-716.
[65] Leyson G P M, Curtin W A, Hector Jr L G, et al. Quantitative prediction of solute strengthening in aluminium alloys[J]. Nature materials, 2010, 9(9): 750-755.

Reviewer: 3

This study offers significant insights into addressing the challenges associated with conventional metal additive manufacturing, such as the high operational and maintenance costs and the narrow processing window imposed by the requirement for high-vacuum or ultra-high-purity inert gas atmospheres. For the first time, the authors proactively introduced readily available and inexpensive trace air into the build chamber during the AM process. By precisely controlling the air content, they achieved tailored microstructural characteristics and mechanical properties, resulting in an exceptional synergy between strength and ductility. A newly developed ML algorithm coupled with APT uncovers two dominant OICs structures and clarifies their role in deformation. The work is highly innovative and opens a fresh avenue for both materials science and AM. I recommend publication in Nature Communications after major revision.

Reply: We are gratified by the reviewer's positive assessment of our work and find their supportive feedback most encouraging.

1. Figure 1(a) clearly shows that, compared to both the as-cast condition and the alloy printed under high-purity argon (Ar-AM), the alloy printed with the optimal air doping content (AD3-AM) achieves an exceptional synergistic improvement in both strength and ductility. Here, a question arises: why does the performance of Ar-AM exceed that of the as-cast alloy? and why is AD4-AM so brittle? Please explain.

Reply: We thank the reviewer for raising this important point regarding the performance comparison. The higher strength of the Ar-AM sample (686 MPa) relative to the as-cast alloy (600 MPa) primarily stems from solid-solution strengthening imparted by interstitial impurities unavoidably introduced during printing. While both materials possess a single BCC phase and similar grain sizes along the tensile direction (as-cast: ~81 μm; Ar-AM short axis: ~95 μm, **Fig. R3**), chemical analysis reveals a key difference: the Ar-AM sample contains higher interstitial contents (0.089 wt% O, 0.030 wt% N) than the high-purity as-cast alloy (0.030 wt% O, 0.004 wt% N), contributing an estimated strengthening increment of ≈45 MPa. Although the oxygen and nitrogen contents measured by chemical analysis methods have noticeable errors, especially at very low concentrations, the trend of solid solution strengthening remains clear.

In contrast, the extreme brittleness of AD4-AM is attributed to the detrimental segregation of excess O and N at grain boundaries [*Jiao M, Lei Z, Wu Y, et al. Manipulating the ordered oxygen complexes to achieve high strength and ductility in medium-entropy alloys[J]. Nature Communications, 2023, 14(1): 806. Chong Y, Gholizadeh R, Tsuru T, et al. Grain refinement in titanium prevents low temperature oxygen embrittlement[J]. Nature communications, 2023, 14(1): 404.*]. APT analysis on a specimen containing a grain boundary (**Fig. R4a**) clearly shows oxygen enrichment at the boundary, and the corresponding 1D concentration profile (**Fig. R4b**) further

confirms noticeable O and N segregation. This excessive interfacial accumulation weakens grain-boundary cohesion and promotes brittle fracture.

To comply with the reviewer's comments, we have revised the manuscript as follows:

Manuscript (Page 5 lines 2-4)

The higher strength of the as-printed (Ar-AM) alloy over its cast counterpart stems from solid-solution strengthening by unintentionally incorporated interstitials: Ar-AM contains 0.089 wt % O and 0.030 wt % N, versus 0.030 wt % O and 0.004 wt % N in the as-cast material.

Manuscript (Page 5 lines 14-15)

as directly evidenced by APT (see Supplementary Fig. 5).

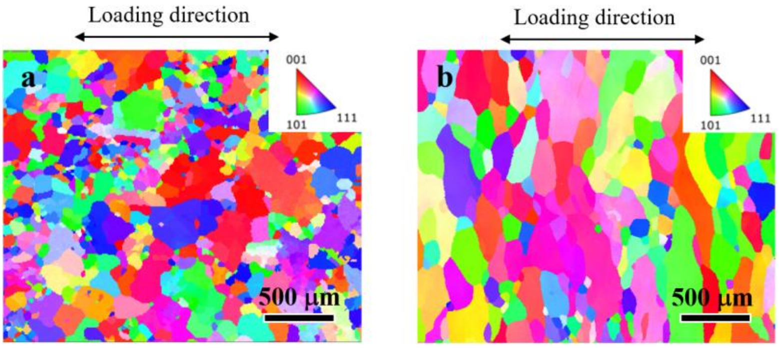

Fig. R3 EBSD IPF maps of (a) as-cast and (b) Ar-AM samples.

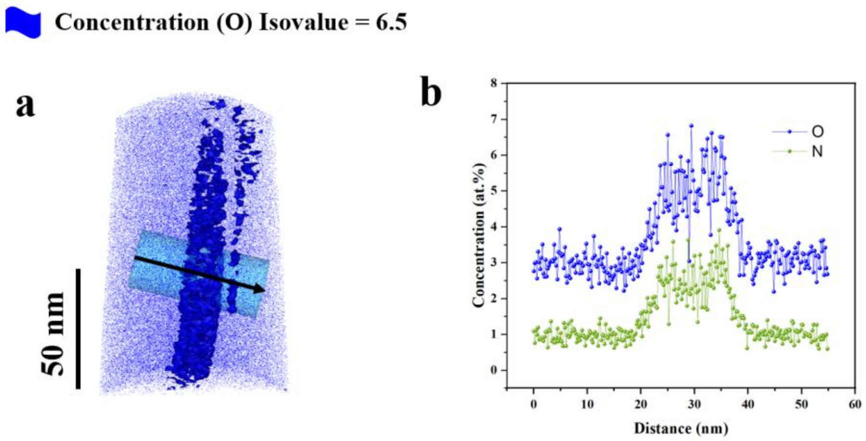

Fig. R4 a, Oxygen isoconcentration surface of 6.5% in the APT specimen containing a grain boundary. **b**, 1D concentration profile along the position marked by the black arrow in (a).

2. When emphasizing the anomalous strength-ductility synergy, the authors compared three alloys: the as-cast alloy, the Ar-AM alloy, and the AD3-AM alloy. Although IPF maps illustrating the grain morphology of the Ar-AM and AD3-AM alloys are provided in Fig. 2b and Supplementary Fig. 8a, respectively, the IPF map of

the as-cast alloy is missing and should be supplemented. Additionally, the grain size distribution should also be provided to allow for a more intuitive understanding of the macroscopic microstructural characteristics.

Reply: We thank the reviewer for the constructive suggestion. Following the recommendation, we have supplemented the microstructure characterization for all three key conditions. As shown in **Fig. R5**, IPF maps and corresponding grain-size distribution histograms are now provided for the as-cast sample, and grain-size distribution histograms are included for both the Ar-AM and AD3-AM samples. The as-cast alloy exhibits equiaxed grains averaging ~81 μm, whereas both additively manufactured counterparts display columnar growth driven by the deposition thermal gradient; however, their aspect ratios remain modest. The Ar-AM and AD3-AM samples have similar long-axis grain sizes (~228 μm and ~221 μm, respectively), while their short-axis sizes are ~95 μm and ~71 μm, respectively. Since the grain dimensions along the tensile direction are comparable across the three conditions, the contribution of grain-refinement strengthening is minimal. Therefore, the exceptional strength-ductility synergy observed in AD3-AM can be attributed primarily to the O and N interstitials.

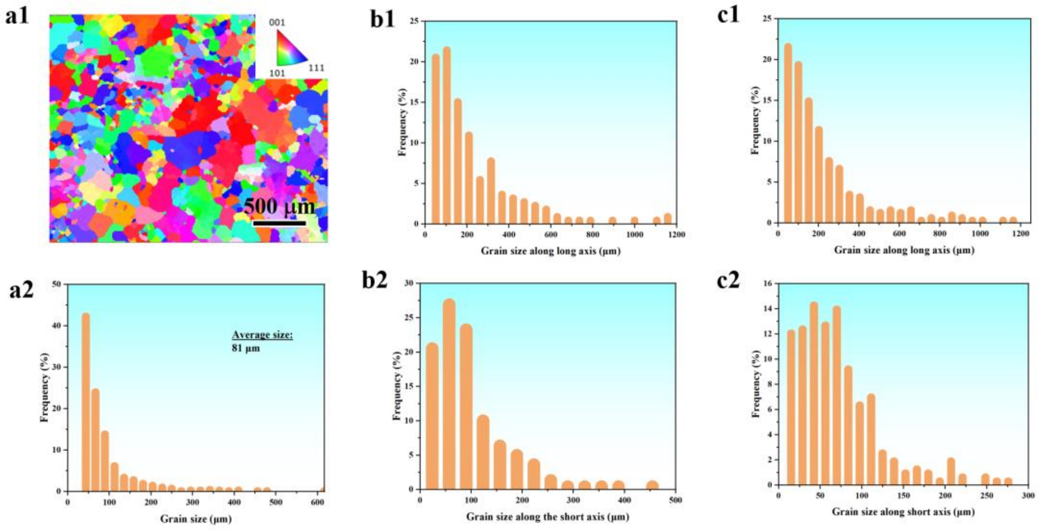

Fig. R5 Microstructure characterization of the as-cast and as-printed samples. a1, IPF map of the as-cast sample. **a2**, Corresponding grain-size distribution histogram. **b1** and **b2**, Grain-size distribution histograms of the Ar-AM sample along the longitudinal and transverse axes, respectively. **c1** and **c2**, Grain-size distribution histograms of the AD3-AM sample along the longitudinal and transverse axes, respectively.

To comply with the reviewer's comments, we have revised the manuscript as follows:

Manuscript (Page 7 lines 8-11)

Supplementary Fig. 16 provides the IPF map of the as-cast alloy and the grain-size distribution histograms for all three key samples. In contrast to the equiaxed

microstructure of the as-cast condition, the AM samples exhibit a discernible but limited difference in grain morphology.

3. Recently, some studies have reported on oxygen addition in additively manufactured titanium alloys [Song T, Chen Z, Cui X, et al. Strong and ductile titanium-oxygen-iron alloys by additive manufacturing. Nature, 2023, 618(7963): 63-68]. While the effectiveness of actively utilizing air for performance tuning in this work is evident, how does this atmosphere-mediated impurity functionalization approach compare with the route of directly adding oxide powders (such as TiO2, ZrO2, etc.) during printing in terms of mechanical properties? Which one is superior? It is recommended that the authors delve into the advantages of the new pathway from a processing perspective, providing an in-depth discussion with clear logic and reasoning.

Reply: We thank the reviewer for raising this insightful comparative question. While direct addition of oxide powders can enhance strength *[Kondoh K, Ichikawa E, Issariyapat A, et al. Tensile property enhancement by oxygen solutes in selectively laser melted titanium materials fabricated from pre-mixed pure Ti and TiO2 powder[J]. Materials Science and Engineering: A, 2020, 795: 139983. Strong and ductile titanium-oxygen-iron alloys by additive manufacturing[J]. Nature, 2023, 618(7963): 63-68.*], our findings suggest the atmosphere-mediated route offers superior property synergy, particularly in retaining ductility. To directly compare the two pathways, we introduced TiO_2 powder onto the TiZrNb matrix powder via ball milling (**Fig. R6a**). After printing, TiO_2 peaks disappeared in the XRD pattern (**Fig. R6b**), indicating decomposition during processing. At 1 at.% O, the yield strength exceeded 800 MPa but ductility remained low. At 2 at.% O, strength rose above 900 MPa, but ductility plummeted below 2%, with fractography revealing brittle intergranular fracture (**Fig. R6c-f**). In contrast, the air-doped AD3-AM sample achieves a yield strength >1 GPa while maintaining >18% tensile ductility, and exhibits a ductile, dimpled fracture surface (**Supplementary Fig. 22**).

The underlying mechanism lies in microstructural homogeneity. In the oxide-doped sample, TEM reveals continuous Ti_2ZrO nanolayers (~10 nm thick) at grain boundaries (**Fig. 7**), providing brittle pathways for crack propagation [1,2]. This phenomenon arises from the combination of rapid solidification and the intrinsic sluggish diffusion of the medium-entropy alloy. These impede the uniform distribution of oxygen, leading to its localized enrichment at grain boundaries where it kinetically favors the formation of continuous brittle nanolayers. Therefore, while both methods increase strength, the atmosphere-mediated approach uniquely avoids harmful grain-boundary phases, thereby preserving ductility and achieving a superior strength-ductility synergy.

In response to the reviewer’s suggestions, we have revised the manuscript as follows:

Manuscript (Page 5 lines 15-17)

It should be emphasized that the alloy printed with TiO_2 addition exhibits significantly inferior performance to its counterpart fabricated via the atmosphere-mediated strategy (see Supplementary Note 3 and Supplementary Figs. 6,7).

Supplementary Information (Page 2 lines 5-6)

Supplementary Note 3. Mechanical and microstructural evaluation of oxide-doped AM TiZrNb alloys

Supplementary Information (Page 3 lines 37 to Page 4 lines 17)

Supplementary Note 3 Mechanical and microstructural evaluation of oxide-doped AM TiZrNb alloys

To directly compare the two pathways, we introduced TiO_2 powder into the TiZrNb matrix powder by ball milling at 100 rpm for 600 min under Ar atmosphere (Supplementary Fig. 6a). The printing parameters remained identical to those used for the AD-AM samples. After printing, TiO_2 peaks disappeared in the XRD pattern (Supplementary Fig. 6b), indicating decomposition during processing. At 1 at.% O, the yield strength exceeded 800 MPa but ductility remained low. At 2 at.% O, strength rose above 900 MPa, but ductility plummeted below 2%, with fractography revealing brittle intergranular fracture (Supplementary Fig. 6c-f). In contrast, the air-doped AD3-AM sample achieves a yield strength >1 GPa while maintaining >18% tensile ductility, and exhibits a ductile, dimpled fracture surface.

The underlying mechanism lies in microstructural homogeneity. In the oxide-doped sample, TEM reveals continuous Ti_2ZrO nanolayers (~10 nm thick) at grain boundaries (Supplementary Fig. 7), providing brittle pathways for crack propagation [1,2]. This phenomenon arises from the combination of rapid solidification and the intrinsic sluggish diffusion of the medium-entropy alloy. These impede the uniform distribution of oxygen, leading to its localized enrichment at grain boundaries where it kinetically favors the formation of continuous brittle nanolayers. Therefore, while both methods increase strength, the atmosphere-mediated approach uniquely avoids harmful grain-boundary phases, thereby preserving ductility and achieving a superior strength-ductility synergy.

Reference in the supplementary information

[1] Neumann G, Tuijn C. Self-diffusion and impurity diffusion in pure metals: handbook of experimental data[M]. Elsevier, 2011.

[2] Xia C, Shi Y, Li Y, et al. Study on microstructure and mechanical and corrosion properties of Zr-Ti-O ternary alloy[J]. Journal of Alloys and Compounds, 2025: 182053.

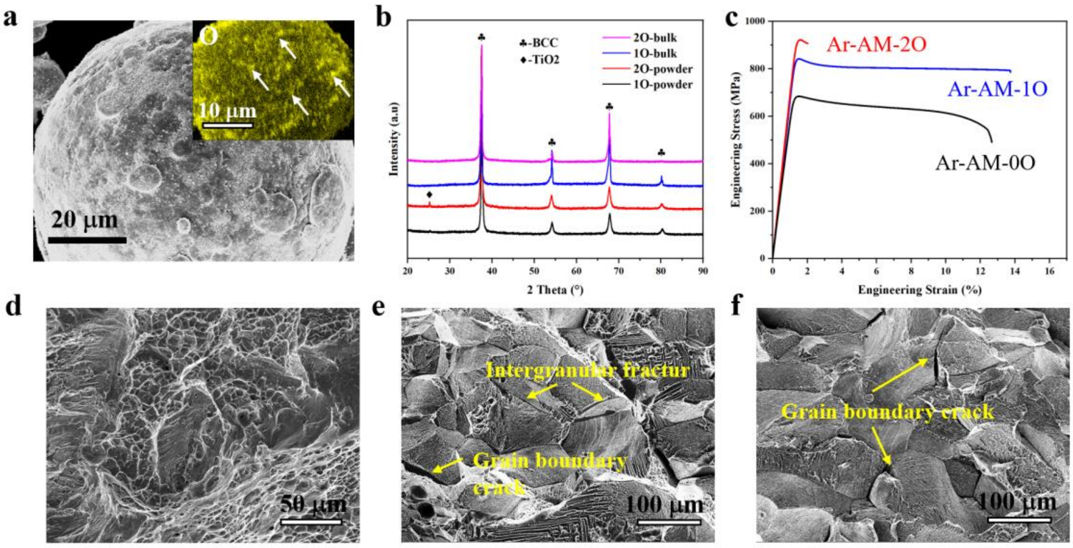

Fig. R6 a, Morphology of the TiZrNb alloy powder after surface coating with TiO_2 powder (the inset shows the EDS mapping of O element; the particle indicated by the white arrow corresponds to TiO_2 powder). **b**, XRD patterns of the alloy powders doped with 1 at.% and 2 at.% O, and the XRD patterns of the corresponding alloys after additive manufacturing. **c**, Engineering stress-strain curves of the base alloy (Ar-AM-0O) fabricated by additive manufacturing, compared with those of the Ar-AM-1O and Ar-AM-2O alloys. **d-f**, Fracture surface morphologies of the Ar-AM-0O, Ar-AM-1O, and Ar-AM-2O alloys, respectively.

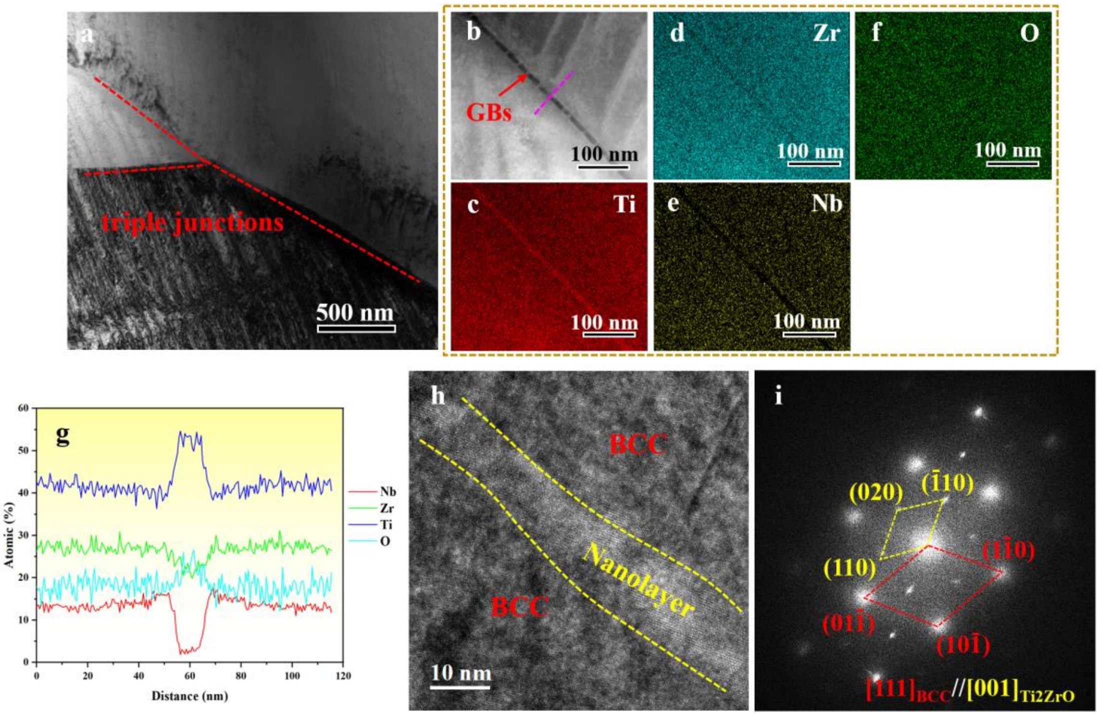

Fig. R7 a, TEM image showing a triple junction. **b**, Magnified view of the grain boundary region marked in (a). **c-f**, Corresponding EDS elemental mapping of the area marked in (b). **g**, Line-scan profile across the grain boundary. **h**, HRTEM image of the grain boundary shown in (b). **i**, SAED pattern taken from the blue square region in (h).

4. The absence of cracks in the alloy after printing with air doping demonstrates its excellent printability. However, porosity also significantly impacts mechanical properties. Although the authors mention that the bulk relative density measured by CT reaches 99.99%, the size and distribution of pores were not provided. It is therefore recommended that the authors supply this important information.

Reply: We thank the reviewer for the positive feedback on printability and the important point regarding porosity. In response, we have performed a quantitative analysis of pore size and distribution from micro-CT data. As shown in **Fig. R8**, the pores follow a normal distribution with an average diameter of 25.9 µm.

Following the reviewer's advice, the following changes have been incorporated into the manuscript:

Manuscript (Page 4 lines 15-17)

The normally distributed 25.9 µm pores observed here represent a typical porosity profile common to AM alloys (Supplementary Fig. 1h).

Supplementary Information (Page 7 lines 6)

h, Pore distribution map of the AD3-AM sample.

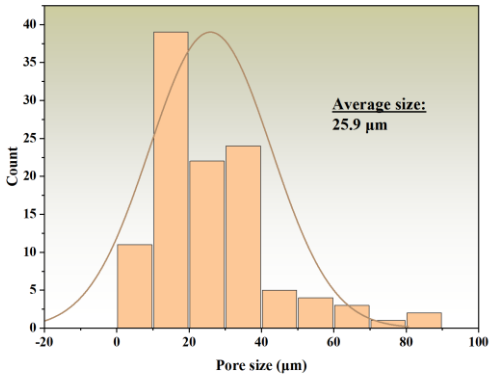

Fig. R8 Pore distribution map of the AD3-AM sample.

5. The repeated thermal cycling effect indeed decomposes the initially surface contamination films into oxygen and nitrogen atoms in solid solution, which represents a distinct advantage of the additive manufacturing process. However, are these elements completely dissolved within the grains, or is there some segregation at the grain boundaries? Oxygen and nitrogen atoms are uniformly distributed at the surface and inside the molten pool? It is well-known that impurity segregation at grain boundaries and the surface of the molten pool often has detrimental effects on mechanical properties. The authors are encouraged to provide high-resolution TEM images or atom probe tomography (APT) data of the grain boundaries to verify this.

Reply: We thank the reviewer for raising this important point regarding the distribution of O and N atoms. Our detailed TEM and APT analyses confirm that for samples within

the optimal doping window (e.g., AD3-AM), the interstitial O and N originating from the controlled atmosphere are uniformly dissolved in the matrix solid solution. This is facilitated by the repeated thermal cycling during AM, which promotes sufficient diffusion, thereby preventing segregation at grain boundaries and avoiding the formation of oxides/nitrides. As shown in **Fig. R9a-c**, no secondary phases are detected along grain boundaries. Moreover, **Fig. R9d-h** shows no noticeable enrichment of O or N at these interfaces. APT analyses further confirm a homogeneous distribution, with no discernible segregation at either the melt-pool surface or its center (**Fig. R10**).

This uniform distribution is crucial for the formation of beneficial OICs within the grains. However, it is important to note that this homogeneous state is achieved only within an optimal doping range. When the doping level becomes excessive (e.g., AD4-AM), pronounced segregation of O and N at grain boundaries occurs, as directly evidenced by APT in **Fig. R4**. This transition explains the embrittlement observed at the highest doping level, underscoring that the exceptional properties are tied to a precisely controlled, uniformly dissolved interstitial state.

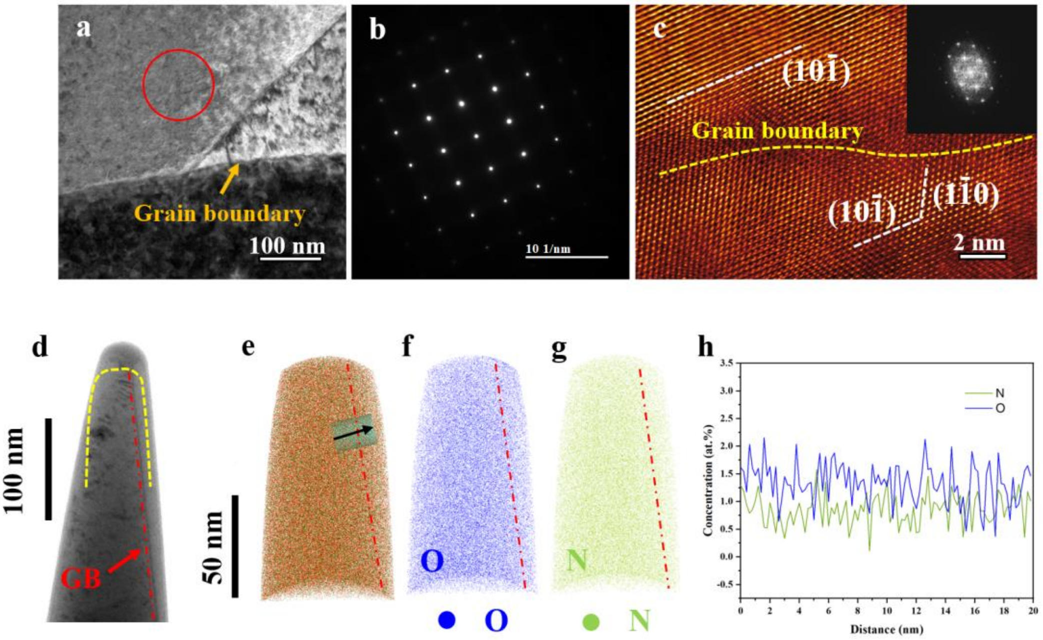

Fig. R9 a, TEM morphology image of the AD3-AM sample containing a triple junction. **b**, SAED pattern corresponding to the red circle in (a). **c**, High-resolution atomic image of the grain boundary. **d**, TEM morphology of the APT tip specimen containing a grain boundary. **e,** 3D atom probe reconstruction of the analyzed volume. **e**, Three-dimensional reconstruction from APT. **f**, **g**, Elemental maps of O and N, respectively. **h**, One-dimensional concentration profile along the grain boundary (indicated by the black arrow in e).

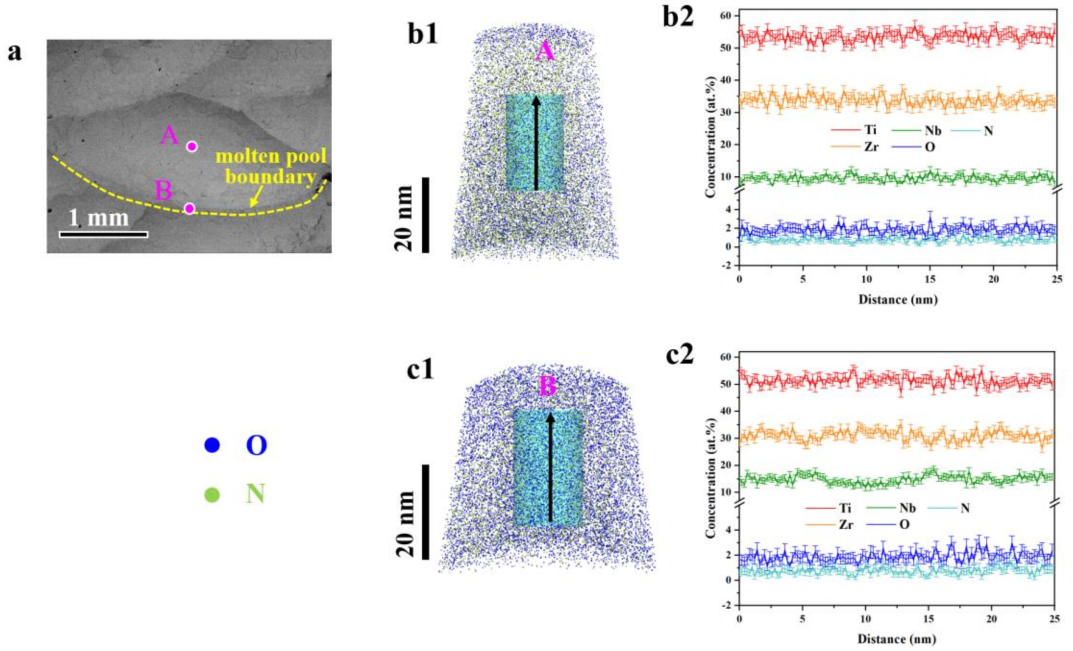

Fig. R10 a, SEM image showing the interface morphology of the AD3-AM sample (the melt pool boundary is outlined by the yellow curve). **b1**, APT reconstruction of O/N elements from region A (melt pool center marked in a); **b2**, corresponding 1D concentration profile across the cylinder in b1. **c1**, APT reconstruction of O/N elements from region B (melt pool surface marked in a); **c2**, corresponding 1D concentration profile across the cylinder in c1.

To address the reviewer's comments, we have made the following amendments:

Manuscript (Page 8 lines 9-12)

It should be noted that the O and N atoms introduced during AM are uniformly dissolved in the matrix, with no detectable segregation at grain boundaries or melt-pool surfaces, and no formation of oxides or nitrides (Supplementary Figs. 15, 16).

6. The authors state in the main text: "The AD3-AM MEA meets all three requirements: its breakdown potential reaches 8 V, far surpassing conventional Ti alloys". It is indeed remarkable to demonstrate such corrosion resistance alongside a yield strength exceeding 1 GPa. However, could the authors supplement this claim with a performance comparison plot against conventional titanium alloys to make the argument more compelling?

Reply: We totally agree that a direct performance comparison would help contextualize our findings. We now provide a direct performance benchmark: the AD3-AM MEA withstands pitting beyond 8 V (**Fig. R11**), surpassing conventional titanium alloys and most advanced grades reported to date (usually <2 V) [27-40]. Although absolute corrosion rates vary with electrolyte, our 3.5 wt % NaCl tests—more aggressive than typical biomedical evaluations—deliver an exceptionally low icorr of 0.114μA/cm^2 while the alloy retains a yield strength above 1 GPa (**Fig. R12**) [41-53]. This unique property profile strongly supports its potential for demanding biomedical applications.

In the revised manuscript, we have added these two figures (**Figs. R11** and **R12**) to provide the requested performance comparison.

Manuscript (Page 7 lines 4)

Supplementary Figs. 9-11

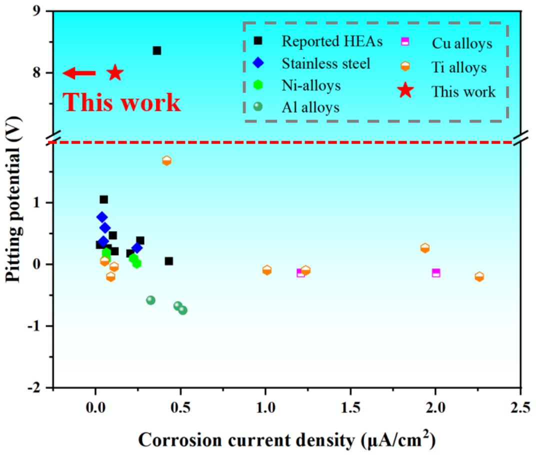

Fig. R11 Comparison of corrosion performance. Pitting potential versus corrosion current density for the AD3-AM medium-entropy alloy, alongside previously reported high-entropy alloys and conventional passive alloys, measured in a 3.5 wt% NaCl solution [27-40].

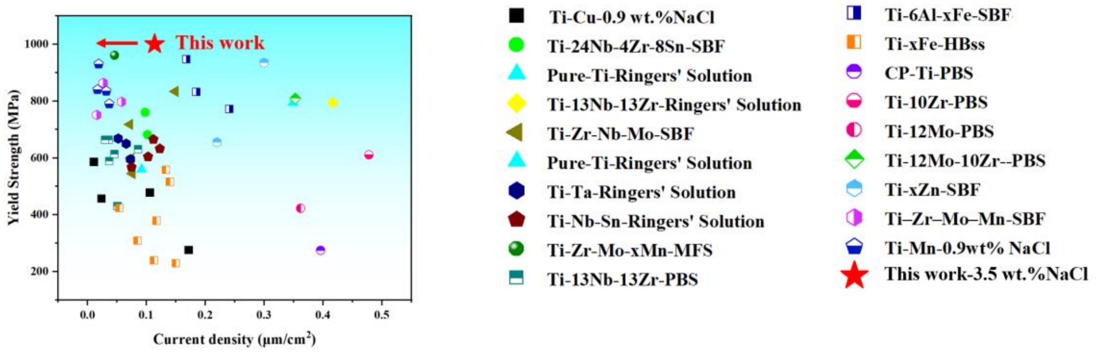

Fig. R12 Comparison of the corrosion current density and yield strength of the AD3-AM medium-entropy alloy with those of most reported titanium alloys. Note that the reference data were obtained from different media and solution concentrations [41-53].

Reference in the supplementary information

[22] Qiu Y, Thomas S, Gibson M A, et al. Corrosion of high entropy alloys[J]. npj Materials degradation, 2017, 1(1): 15.

[23] Shi Y, Yang B, Liaw P K. Corrosion-resistant high-entropy alloys: a review. Metals 7: 43[EB/OL].(2017).

[24] Shi Y, Yang B, Xie X, et al. Corrosion of AlxCoCrFeNi high-entropy alloys: Al-content and potential scan-rate dependent pitting behavior[J]. Corrosion Science, 2017, 119: 33-45.

[25] Lin C M, Tsai H L. Evolution of microstructure, hardness, and corrosion properties of high-entropy Al0.5CoCrFeNi alloy[J]. Intermetallics, 2011, 19(3): 288-294.

[26] Hsu Y J, Chiang W C, Wu J K. Corrosion behavior of FeCoNiCrCux high-entropy alloys in 3.5% sodium chloride solution[J]. Materials Chemistry and Physics, 2005, 92(1): 112-117.
[27] Wu C L, Zhang S, Zhang C H, et al. Phase evolution and cavitation erosion-corrosion behavior of FeCoCrAlNiTix high entropy alloy coatings on 304 stainless steel by laser surface alloying[J]. Journal of Alloys and Compounds, 2017, 698: 761-770.
[28] Zhang S, Wu C L, Zhang C H, et al. Laser surface alloying of FeCoCrAlNi high-entropy alloy on 304 stainless steel to enhance corrosion and cavitation erosion resistance[J]. Optics & Laser Technology, 2016, 84: 23-31.
[29] Shang C, Axinte E, Sun J, et al. CoCrFeNi(W1-xMox) high-entropy alloy coatings with excellent mechanical properties and corrosion resistance prepared by mechanical alloying and hot pressing sintering[J]. Materials & Design, 2017, 117: 193-202.
[30] Ye Q, Feng K, Li Z, et al. Microstructure and corrosion properties of CrMnFeCoNi high entropy alloy coating[J]. Applied Surface Science, 2017, 396: 1420-1426.
[31] Qiu X. Microstructure, hardness and corrosion resistance of Al2CoCrCuFeNiTix high-entropy alloy coatings prepared by rapid solidification[J]. Journal of Alloys and Compounds, 2018, 735: 359-364.
[32] shankar Dhandapani V, Thangavel E, Arumugam M, et al. Effect of Ag content on the microstructure, tribological and corrosion properties of amorphous carbon coatings on 316L SS[J]. Surface and Coatings Technology, 2014, 240: 128-136.
[33] Qiu Y, Gibson M A, Fraser H L, et al. Corrosion characteristics of high entropy alloys[J]. Materials science and technology, 2015, 31(10): 1235-1243.
[34] Pardo A, Merino M C, Coy A E, et al. Pitting corrosion behaviour of austenitic stainless steels–combining effects of Mn and Mo additions[J]. Corrosion Science, 2008, 50(6): 1796-1806.
[35] Zhou Q, Sheikh S, Ou P, et al. Corrosion behavior of Hf0.5Nb0.5Ta0.5Ti1.5Zr refractory high-entropy in aqueous chloride solutions[J]. Electrochemistry Communications, 2019, 98: 63-68.
[36] Wang J, Zhang S, Sun Z, et al. Optimization of mechanical property, antibacterial property and corrosion resistance of Ti-Cu alloy for dental implant[J]. Journal of materials science & technology, 2019, 35(10): 2336-2344.
[37] Li X, Tang J, Ju J, et al. Superior corrosion resistance and good biocompatibility of Ti-24Nb-4Zr-8Sn alloy fabricated by a cost-effective, net-shape powder metallurgy method[J]. Materials Futures, 2025, 4(3): 035401.
[38] Zhou L, Yuan T, Tang J, et al. Mechanical and corrosion behavior of titanium alloys additively manufactured by selective laser melting-A comparison between nearly β titanium, α titanium and α+ β titanium[J]. Optics & Laser Technology, 2019, 119: 105625.
[39] Chui P, Jing R, Zhang F, et al. Mechanical properties and corrosion behavior of β-type Ti-Zr-Nb-Mo alloys for biomedical application[J]. Journal of Alloys and Compounds, 2020, 842: 155693.
[40] Zhao D, Han C, Li Y, et al. Improvement on mechanical properties and corrosion resistance of titanium-tantalum alloys in-situ fabricated via selective laser melting[J]. Journal of Alloys and Compounds, 2019, 804: 288-298.
[41] Liu H, Yang J, Zhao X, et al. Microstructure, mechanical properties and corrosion behaviors of biomedical Ti-Zr-Mo-xMn alloys for dental application[J]. Corrosion Science, 2019, 161: 108195.

[42] Hariharan A, Goldberg P, Gustmann T, et al. Designing the microstructural constituents of an additively manufactured near β Ti alloy for an enhanced mechanical and corrosion response[J]. Materials & Design, 2022, 217: 110618.
[43] Lu J, Zhao Y, Niu H, et al. Electrochemical corrosion behavior and elasticity properties of Ti-6Al-xFe alloys for biomedical applications[J]. Materials Science and Engineering: C, 2016, 62: 36-44.
[44] Niu J, Guo Y, Li K, et al. Improved mechanical, bio-corrosion properties and in vitro cell responses of Ti-Fe alloys as candidate dental implants[J]. Materials Science and Engineering: C, 2021, 122: 111917.
[45] Guo Z, Huang Y, Sun C, et al. Ti-Mo-Zr alloys for bone repair: mechanical properties, corrosion resistance, and biological performance[J]. journal of materials research and technology, 2023, 24: 7624-7637.
[46] Chen Y T, Xu J L, Huang J, et al. Microstructure, mechanical properties, and corrosion resistance of biomedical Ti-Zn alloys prepared by spark plasma sintering[J]. Intermetallics, 2025, 185: 108926.
[47] Li Z, Wo J, Fu Y, et al. Effects of Zr addition on the microstructural evolution, mechanical properties, and corrosion behavior of novel biomedical Ti-Zr-Mo-Mn alloys[J]. ACS Biomaterials Science & Engineering, 2023, 9(12): 6935-6946.
[48] Zhou X, Fang H, Yuan T, et al. Effect of slight Sn modification on mechanical properties and corrosion behavior of Ti-(2-4 wt%) Mn alloys fabricated via powder metallurgy[J]. Materials Characterization, 2023, 203: 113068.

7. The repeated thermal cycling process of AM also results in differences in microstructures and mechanical properties at different heights. How in this work? In addition, anisotropic microstructure and mechanical properties are common during the AM. they can be considered.

Reply: We thank the reviewer for raising these critical points regarding thermal-cycle-induced gradients and anisotropy in AM. The inherent repeated thermal cycling in additive manufacturing can indeed lead to variations in mechanical properties along the build height in some metastable materials [*Zhang J, Liu Y, Sha G, et al. Designing against phase and property heterogeneities in additively manufactured titanium alloys[J]. Nature communications, 2022, 13(1): 4660. Zhang J, Bermingham M J, Otte J, et al. Ultrauniform, strong, and ductile 3D-printed titanium alloy through bifunctional alloy design[J]. Science, 2024, 383(6683): 639-645.*].

To evaluate potential variations along the build height, we fabricated a bulk sample and extracted tensile specimens from four different heights (H1-H4, ~4 mm interval) along the building direction (XY plane) (**Fig. R13a**). The tensile specimens had a gauge length of 8 mm and a thickness of 1 mm. Tensile curves (**Fig. R13b**) indicate that both yield strength and ductility show minor, stochastic fluctuations without a height-dependent trend. Unlike metastable titanium alloys, the TiZrNb alloy retains a stable BCC structure; consequently, despite unavoidable random scatter, property variations along the build height remain insignificant. Concurrently, we explicitly characterized the in-plane anisotropy. Specimens were extracted from the YZ/XZ planes to test the

“horizontal” (perpendicular to deposition but parallel to scan track) and “vertical” (parallel to deposition) directions. The tensile results (**Fig. R13c**) reveal obvious anisotropy: the horizontal direction exhibits higher strength and ductility, while the vertical (building) direction shows the lowest performance.

The anisotropy is directly linked to microstructural and defect features. For samples from the XY plane, the applied stress first encounters the relatively weak boundaries between adjacent laser scan tracks. Corresponding fracture surfaces exhibit shallow dimples with internal micro-voids, and side surfaces reveal micro-cracks perpendicular to the loading direction (**Fig. R14a1-a4**). These defects detrimentally affect both strength and ductility [*Wolff S, Lee T, Faierson E, et al. Anisotropic properties of directed energy deposition (DED)-processed Ti-6Al-4V[J]. Journal of Manufacturing Processes, 2016, 24: 397-405.*]. In the YZ-plane (horizontal) specimens the tensile axis runs parallel to the scan tracks and hence lies within the strongly bonded melt-pool interiors; simultaneously, it coincides with the short-axis direction of the columnar grains, so the higher grain-boundary density provides extra grain-size strengthening. These combined effects produce fracture surfaces covered with deep dimples and side surfaces decorated with wavy slip lines that attest to extensive cross-slip (**Fig. R14b1-b4**), accounting for the simultaneously high strength and ductility observed in this orientation. However, the property degradation observed along the building direction (XZ plane, vertically) is attributed to weak interlayer interfaces [*Gou S, Gao M, Shi Y, et al. Additive manufacturing of ductile refractory high-entropy alloys via phase engineering[J]. Acta Materialia, 2023, 248: 118781.*]. Their side surfaces display numerous load-perpendicular cracks, and the failure mode is quasi-brittle (**Fig. R14c1-c4**).

IPF maps from the top to the bottom (**Fig. R 15**) reveal a transition from columnar grains at the top to a more equiaxed morphology at the bottom. This is attributed to repeated thermal cycling in the lower regions, which provides additional driving force for grain boundary migration and microstructural homogenization. The influence of microstructural and defect characteristics (morphology, size and distribution) on tensile properties in AM alloys is extremely complex [*Wolff S, Lee T, Faierson E, et al. Anisotropic properties of directed energy deposition (DED)-processed Ti-6Al-4V[J]. Journal of Manufacturing Processes, 2016, 24: 397-405. Huangfu B, Liu Y, Liu X, et al. Anisotropy of Additively Manufactured Metallic Materials[J]. Materials, 2024, 17(15): 3653. Kok Y, Tan X P, Wang P, et al. Anisotropy and heterogeneity of microstructure and mechanical properties in metal additive manufacturing: A critical review[J]. Materials & Design, 2018, 139: 565-586.*], and full understanding remains an area for future systematic research.

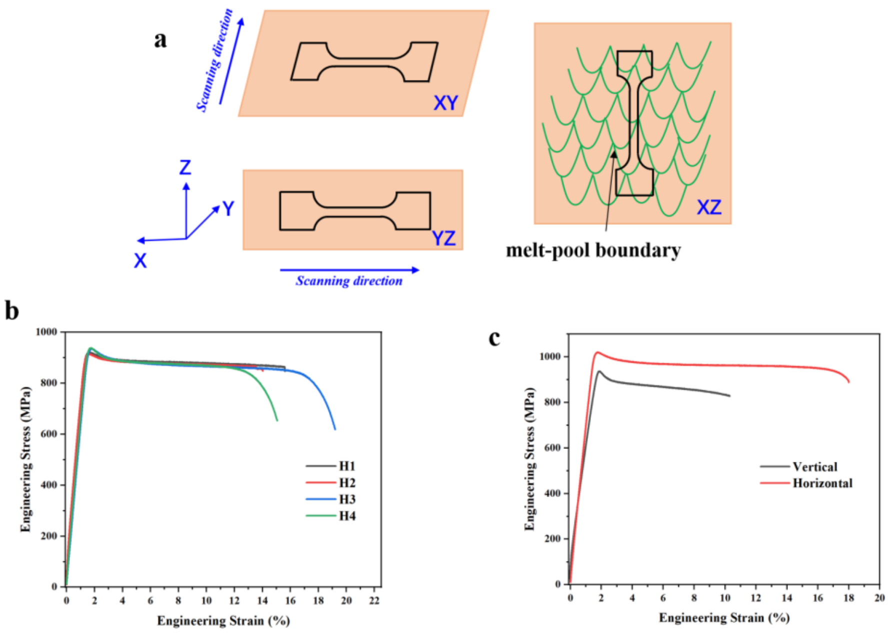

Fig. R13 a, Tensile-specimen extraction protocol. XY-plane coupons (building direction, BD) were machined perpendicular to the scan track; anisotropy was assessed with YZ- and XZ-plane coupons oriented either horizontal (perpendicular to BD but parallel to the scan track) or vertical (parallel to BD). **b**, Engineering stress-strain curves for specimens extracted at sequential heights (H1-H4: approximately 4, 8, 12, and 16 mm below the top surface, respectively). c, Stress-strain curves of the as-deposited sample tested horizontally and vertically.

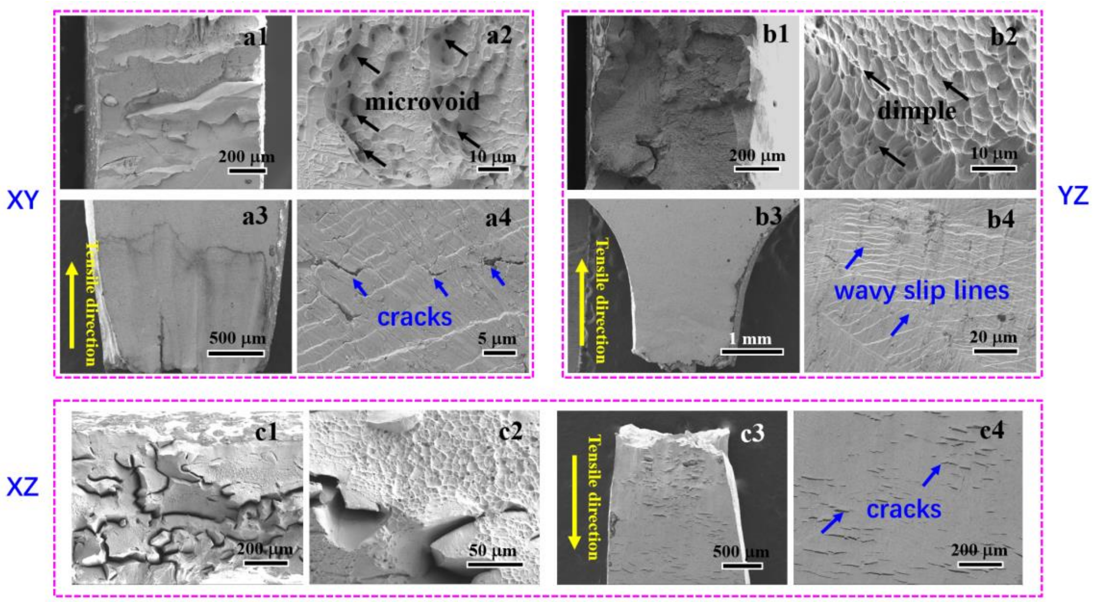

Fig. R14 Post-tensile microstructure. a1-a4, XY-plane view of the deformed specimen: **a1** and **a2**, fracture surface; **a3** and **a4**, side surface. **b1-b4**, Horizontal tensile test (XZ plane): **b1** and **b2**, fracture surface; **b3** and **b4**, side surface. **c1-c4**, Vertical tensile test (XZ plane): **c1** and **c2**, fracture surface; **c3** and **c4**, side surface.

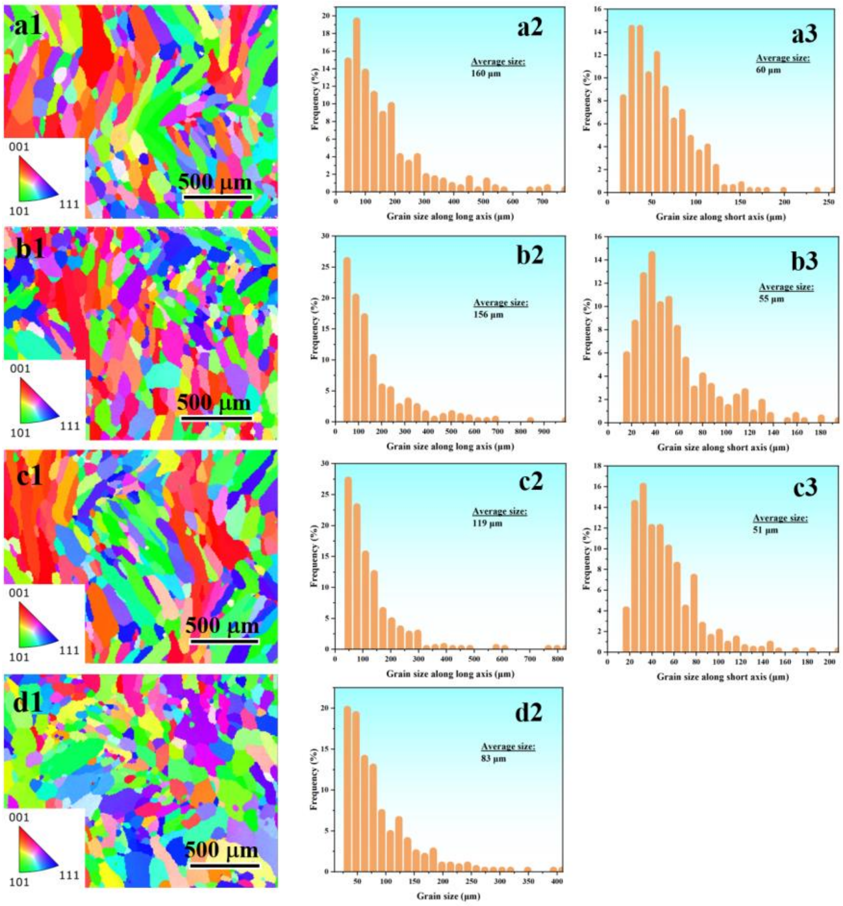

Fig. R15 a1-d1, IPF maps acquired sequentially from top to bottom, all viewed along the building direction. **a2-c2**, Grain-size distributions along the longitudinal axis of maps a1-c1, respectively. **a3-c3**, Grain-size distributions along the transverse axis of maps a1-c1, respectively. **d2**, Average grain-size histogram derived from map d1.

8. How to quantify the content of oxygen and nitrogen dissolved in the alloy?

Reply: We thank the reviewer for raising this important point regarding the quantification of dissolved oxygen and nitrogen. To accurately determine the total oxygen and nitrogen content in the bulk alloys, we utilized a LECO Instruments inert gas fusion (IGF) system equipped with infrared (IR) detection. This method quantitatively measures the bulk average concentration by melting the sample in a flowing inert gas stream and detecting the released gases via IR absorption. **Table R1** and **Fig. R16** present the absorbed oxygen and nitrogen contents in the as-printed alloys under various air-doping levels.

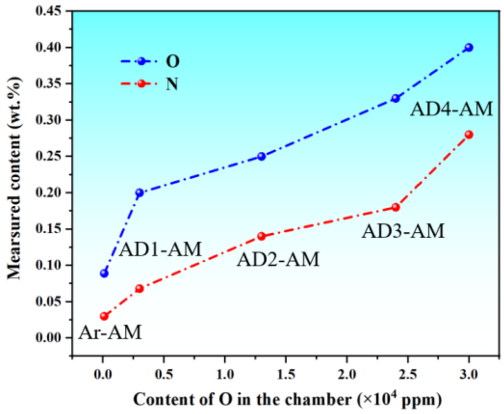

Fig. R16 Oxygen and nitrogen absorption under different processing conditions. Plots showing the absorbed O and N contents in the samples, correlated with the oxygen levels recorded by the in-situ sensor during printing.

Point-by-point responses

Reviewer #3 (Remarks to the Author):

This work holds significance for the additive manufacturing of metallic parts and broadens the processing window. All comments have been addressed, and the manuscript quality meets the standards for publication.

Reply: We are grateful for the reviewers' positive feedback and their recommendation to publish our work in Nature Communications. Furthermore, we extend our sincere gratitude to the reviewers for their earlier constructive comments, which have proven invaluable in refining and strengthening our manuscript.
